# Multi-omics characterization of a scoring system to quantify hypoxia patterns in patients with head and neck squamous cell carcinoma

**DOI:** 10.1186/s12967-022-03869-8

**Published:** 2023-01-10

**Authors:** Cong Peng, Huiping Ye, Zhengyang li, Xiaofeng Duan, Wen Yang, Zhuguang Yi

**Affiliations:** 1 Department of Otolaryngology, Guizhou Provincial People’s Hospital, Guiyang, China; 2 Department of Oral and Maxillofacial Surgery, Guizhou Provincial People’s Hospital, Guiyang, China; 3 Department of Pathology, Affiliated Hospital of Guizhou Medical University, Guiyang, China

**Keywords:** Hypoxia, Head and neck squamous cell carcinoma, Crosstalk, Single-cell, Cellchat

## Abstract

**Background:**

The 5-year survival rate of patients with head and neck squamous cell carcinoma (HNSCC) remains  < 50%. Hypoxia patterns are a hallmark of HNSCC that are associated with its occurrence and progression. However, the precise role of hypoxia during HNSCC, such as the relationship between hypoxia, tumor immune landscape and cell communication orchestration remains largely unknown. The current study integrated data from bulk and single-cell RNA sequencing analyses to define the relationship between hypoxia and HNSCC.

**Methods:**

A scoring system named the hypoxia score (HS) was constructed based on hypoxia-related genes (HRGs) expression. The predictive value of HS response for patient outcomes and different treatments was evaluated. Single-cell datasets and cell communication were utilized to rule out cell populations which hypoxia targeted on.

**Results:**

The survival outcomes, immune/Estimate scores, responses to targeted inhibitors, and chemotherapeutic, and immunotherapy responses were distinct between a high HS group and a low HS group (all *P* < 0.05). Single-cell datasets showed different distributions of HS in immune cell populations (*P* < 0.05). Furthermore, *HLA-DPA1*/*CD4* axis was identified as a unique interaction between CD4 + T Conv and pDC cells.

**Conclusions:**

Altogether, the quantification for hypoxia patterns is a potential biomarker for prognosis, individualized chemotherapeutic and immunotherapy strategies. The portrait of cell communication characteristics over the HNSCC ecosystem enhances the understanding of hypoxia patterns in HNSCC.

**Supplementary Information:**

The online version contains supplementary material available at 10.1186/s12967-022-03869-8.

## Introduction

Head and neck squamous cell carcinoma (HNSCC) is the sixth most commonly diagnosed malignant tumor worldwide. Tumors that arise on the lip, oral cavity, larynx, oropharynx, and hypopharynx accounted for 744,994 cases and 364,339 deaths in 2020, respectively [1]. More than 50% of cases are diagnosed in the advanced stages of the disease [2]. Previous studies have shown that the HNSCC 5-year survival rate is still  < 50% due to the risk of metastasis or recurrence [3]. However, major treatment modalities, including surgery, radiotherapy, chemotherapy, and combination strategies do not effectively improve patient survival and often compromise the complex anatomy of the head and neck, affecting daily functions such as swallowing and articulation and impairing patient quality of life (QOL) [4] Compromised QOL is likely to explain why HNSCC patients have the second-highest rate of suicide (63.4 cases per 100,000 individuals) among cancer patients [5]. While noninvasive immunotherapy shows great potential as a cancer treatment by activating the body’s defense system to eliminate cancer cells, it only works for some patients [6]. A deeper understanding of the molecular and cellular mechanisms underlying HNSCC progression or metastasis and the identification of new targets for cancer immunotherapy is urgently needed.

Hypoxia is a hallmark of the tumor microenvironment (TME) in major human cancer, including HNSCC, regulating tumor growth and metastasis and leading to poor prognosis, resistance to radiation therapy, immune evasion, and immune resistance [7, 8]. Hypoxia-inducible factor 1α(HIF1α) is a subunit of the transcription factor HIF1, which promotes tumor cell survival and helps to reprogram from oxidative phosphorylation to glycolysis of tumors (the Warburg effect)in response to hypoxia [9]. As a result of hypoxia, proinflammatory and immune-modulating cytokines and chemokines are released [10]. On the other hand, hypoxia could reduce the infiltration and activity of CD8 + T cells, dendritic cells (DCs) and natural killer (NK) cells [11]. Due to these features in cancer, the hypoxia-related genes(HRGs) modulate pathway may serve as potential prognostic biomarkers of HNSCC, and targeting these genes may prove helpful to cancer therapy. Multiple studies reported that HRGsare associated with tumor progression, metastasis and tumor sensitivity to treatments, such as colorectal cancer, lung cancer, pancreatic ductal adenocarcinoma, and brain tumors [12–15]. As for HNSCC, Li et al. [16] demonstrated a hypoxia-related genes signatureto predict survival and guide personalized clinical treatment based on 6 HRGs. As cancer is a complex and highly heterogeneous disease that involves a multitude of gene interactions, gene signature models constructed based on multiple HRGs rather than fewer HRGs will contribute to enhancing our understanding of the hypoxia patterns in HNSCC. In addition, single-cell RNA sequencing (scRNA-seq) is a powerful tool to explore single-cell expression patterns in bulk tumor tissue, making it possible to understand immune cellular heterogeneity and the relationship between the microenvironment crosstalk and cancer. However, the relationships among characteristics of hypoxia-related subtypes, tumor immune landscape and cell communication orchestration in the scRNA-seq data remain scanty.

This study established a scoring model to quantify the characteristics of hypoxia and analyzed the relationship between the hypoxia score (HS) and disease prognosis, immune characteristics, and clinical treatment sensitivity. Surprisingly, multi-omics revealed differences between the high HS group (HHSG) and the low HS group (LHSG). In addition, a single-cell sequencing dataset (GSE139324) was used to further elucidate the relationship between hypoxia and HNSCC. Findings could be used to inform the development of individualized treatments and provide new treatment targets.

## Materials and methods

### Data acquisition and processing

**Fig. 1:**
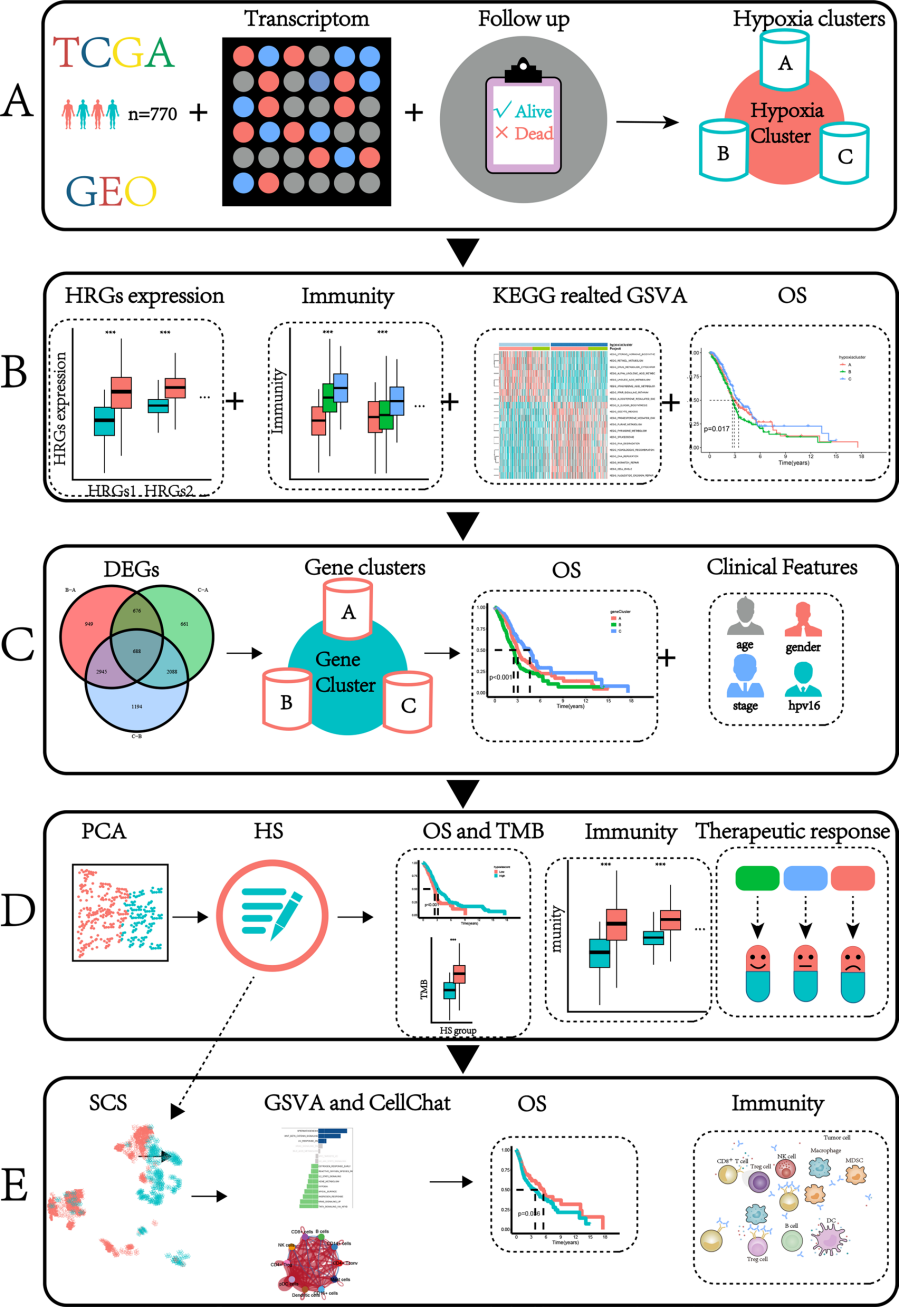
Flowchart of the study. TCGA, The Cancer Genome Atlas; GEO, Gene Expression Omnibus; HRGs, hypoxia-related genes; OS, overall survival; DEGs, differentially expressed genes; GSVA, gene set variation analysis; KEGG, Kyoto Encyclopedia of Genes and Genomes; PCA, Principal Component Analysis; TMB, tumor mutation burden; SCS, single-cell sequence

The data sources and analysis methods used in this study are shown in Fig. 1. Bulk tumor tissue RNAseq and somatic mutation information, copy number variation (CNV), clinical characteristics, and survival data were all obtained from The Cancer Genome Atlas (TCGA)(https://tcga-data.nci.nih.gov/tcga/, accessed 12 June 2022) and Gene Expression Omnibus(GEO) (http://www.ncbi.nlm.nih.gov/geo/, accessed 12 June 2022). A total of 547 samples were collected from TCGA (503 HNSCC patients and 44 normal tissues), and 270 and 97 HNSCC patient samples were obtained from the GSE65858 and GSE41613 datasets, respectively (Table 1 and Table 2). The FPKM value from TCGA was converted to transcripts per kilobase million (TPM) and combined with GSE65858 as a data matrix. After excluding some cases with missing survival time, a total of 770 samples were obtained and used for follow-up and analysis.

**Table 1:** Clinical characteristics of patients with head and neck squamous carcinoma (HNSCC) in the Cancer Genome Atlas (TCGA) dataset and GSE65858

Variable	Number of samples
Age at diagnosis(< 60/ ≥ 60/NA)	418/379/1
Gender (male/female)	608/190
Grade(G1/G2/G3/G4/NA)	63/311/124/7/291
Stage(I/II/III/IV/NA)	27/74/82/269/76
T (T0/T1/T2/T3/T4/NA)	1/49/140/101/174/63
M(M0/M1/NA)	453/8/337
N(N0/N1/N2/N3/NA)	273/100/304/20/101
HPV16(negative/positive/NA)	128/237/433

**Table 2:** Clinical characteristics of patients with HNSCC in the GSE41613 dataset

Variable	Number of samples
Age at diagnosis (< 60/ ≥ 60)	50/47
Gender(Male/Female)	66/31
Stage(I-II/III-IV)	41/56
HPV16(negative)	97

Single-cell RNAseq analysis was conducted on five healthy donors and twenty-six patients with HNSCC from the GEO database (GSE139324) (Table 3). During data integration, ‘harmony’ package(version: 0.1.0) was able to mitigate batch effects [17]. The Expression data were normalized with the ‘NormalizeData’ function in the ‘Seurat’ R package (version: 4.1.1) [18]. The first 2,000 highly variable genes were identified using the ‘FingVariableGenes’ function of the ‘Seurat’ R package. Principal component analysis (PCA) was used to explore the sources of these highly variable genes via the ‘RunPCA’ function of the ‘Seurat’ R package, and cell subpopulations were annotated using the ‘singleR’ R package (version:1.10.0) and the marker genes from the previous study [19]. The distribution of the cellular components was plotted using the ‘TSNE’ R package (version: 0.16) [20]. The crosstalk of different cell subtypes in HNSCC was explored using ‘CellChat’ R package (version:1.5.0) [21].

**Table 3:** Clinical characteristics of patients with HNSCC in the GSE139324 dataset

Variable	Healthy donor	HNSCC
Age (< 60/ ≥ 60)	3/2	13/13
Gender(Male/Female)	3/2	20/6
Stage(I-II/III-IV)	–	12/14
HPV16(negative)	–	18/8

### Unsupervised clustering of hypoxia-related genes

A total of 49 HRGs obtained from the buffa hypoxia metagene dataset in the Molecular Signature Database (MSigDB) were used to identify different hypoxia-related patterns (version 7.4, https://www.gsea-msigdb.org/gsea/msigdb/, accessed 12 June 2022). The sample was clustered using the ‘ConsensusClusterPlus’ R package (version:1.60.0) and the hierarchical clustering method was repeated 1,000 times to ensure cluster stability [22].

### Immune cell infiltration and function

Single-sample gene set enrichment analysis (ssGSEA) was used to quantify immune cell infiltration and function in each sample using the ‘GSVA’ R package (version:1.44.0) [23]. In addition, 48 immune checkpoints were obtained from prior studies [24, 25].Stromal/immune/Estimate scores were calculated with the ‘estimate’ R package (version:1.0.13) [26]. The correlation between the *HLA-DPA1*/*CD4* expression and marker genes of various immune cell types was analyzed using the Tumor Immune Estimation Resource (TIMER) database (https://cistrome.shinyapps.io/timer/, accessed 12 September 2022) [27]. In addition, the correlation between hub genes and immune cell infiltration was assessed using Gene Set Cancer Analysis (GSCA) (http://bioinfo.life.hust.edu.cn/GSCA/#/expression/, accessed 12 September 2022) [28].

### Biofunction prediction

The ‘GSVA’ R package (version:1.44.0) was used to clarify the biological processes of different hypoxia clusters or cell subpopulations [23]. Hypoxia-related pathways were presented as heatmaps in the hypoxia clusters and bar plots of different cell subpopulations (all adjusted *P* < 0.05).

### Hypoxia scoring model

First, three hypoxia clusters were identified based on the expression of 49 HRGs using consensus clustering analysis. Second, differentially expressed genes (DEGs) in the three clusters were retrieved using the ‘limma’ R package (version: 3.52.1). A total of 688 DEGs were obtained after the intersection among the 3 hypoxia clusters (adjusted *P* < 0.001) and evaluated using univariate Cox regression analysis. Of these, 207 DEGs associated with disease prognosis were obtained and used to further analysis (*P* < 0.05). The hypoxia score (HS) of each sample was calculated based on the PCA analysis as follows:

$$\mathrm{HS}=\sum \mathrm{PCA}1\mathrm{ i}+\sum \mathrm{PCA}2\mathrm{ i}$$

‘i’ is the expression of the 207 prognostic DEGs. The HS of each patient in the GSE41613 and each cell in the GSE139324 datasets was designed using a similar formula, respectively.

### Potential sensitive multiple therapeutic predictions

The response of the HHSG and LHSG groups to drug therapy and immunotherapy was compared. The ‘pRRophetic’ R package (version: 0.5) was used to determine the concentration of various chemotherapy, targeted inhibitor, and immunotherapy drugs that caused a 50% reduction (IC50) in growth [29]. Immunophenoscores (IPS) for HNSCC were then downloaded from the Cancer Immunome Atlas (TCIA) (https://tcia.at/, accessed 20 June 2022) [30]. The IPS of the HHSG and LHSG were compared to predict sensitivity to immunotherapy. In addition, the Tumor Immune Dysfunction and Exclusion (TIDE) algorithm (http://tide.dfci.harvard.edu, accessed 20 June 2022) was used to predict HNSCC patient response to immune checkpoint inhibitors [31]. A total of 14 cisplatin-sensitive and 4 cisplatin-resistant HNSCC cell lines from the GSE102787 dataset were used to assess the ability of the HS to predict chemotherapy responses.

### Identification of key genes related to hypoxia

The weighted gene co-expression network analysis (WGCNA) R package (version:1.71)was used to obtain the key prognostic HRGs in the HNSCC [32]. Based on the TCGA-HNSCC gene expression matrix, the suitable power exponent was selected and the adjacency matrix was converted to the topological overlap matrix. Then, the correlation analysis between the gene consensus modules with HS was performed, and the top 2 correlation coefficient modules positively correlated with HS were selected for further analyses. After intersection of the genes obtained from the 2 module and the 49 HRGs and a careful literature search, the key prognostic HRG was identified [30]. The Human Protein Atlas (HPA) database was used to compare key prognostic HRG protein expression between normal and tumor tissue in HNSCC.

### Cell culture and retroviral infection

SCC-4 cells were purchased from BeNa Culture Collection (Xinyang, China) and cultured in 90%Dulbecco’s Modified Eagle Medium/Nutrient Mixture F-12(DMEM-H/F12; Procell Life, China) supplemented with 10% fetal bovine serum (FBS; Procell Life, China) and 400 ng/ml hydrocortisone. The SCC-4 cells were incubated with 5% carbon dioxide at 37 °C, plasmid transfection was carried out using Lipofectamine 3000 reagent (Thermo Fisher Scientific, China).

### Western blotting

As described previously, western blot analysis was performed [33]. In brief, after the total protein was extracted, the BCA protein concentration reagent is quantified and then subjected to SDS-PAGE. A 20 μl protein sample was then transferred to a PVDF membrane, which was repeatedly washed before adding chemiluminescent solution (ECL, Thermo Fisher Scientific, China) to image the protein.

### CCK-8 assay

Cell growth and viability were measured using a Cell Counting Kit-8 (CCK-8) kit (Solarbio, Beijing, China), cells in a 96-well plate, about 2 × 10^3^ cells in each well. After 48 h incubation, 10 μl of CCK-8 was added to each well and incubated at 37 °C for 1 h. The optical density at 450 nm of each well was measured once at 0,24, 48, 72 and 96 h.

### Transwell assays

Cell migration analysis was performed according to the descriptions in a previous study [34]. Briefly, all the groups of cells were first resuspended in medium and the transwell chambers (Corning, USA) were removed after 24 h of incubation, followed by fixation of the invading cells with 4% paraformaldehyde and staining with 0.1% crystalline violet. Finally, transwell chambers were inverted and photographed under a microscope.

### Statistical analyses

All statistical analyses in this study were conducted using R language software (version 4.2.1) (Bell Laboratories, formerly AT&T, now Lucent Technologies). The ‘maftool’ R package (version 2.12.0) and ‘rcircos’ R package (version 1.2.2) were used to draw a waterfall plot of the gene mutation status of different clusters and illustrate the position of the HRG CNV on the chromosomes, respectively [35, 36]. Variables in data analysis including continuous(e.g., HRGs expressions, HS, OS time) and categorical variables(e.g., survival status). Kaplan-Merier curves are presented and the differences in the prognosis of different types of patients between the curves are tested using log-rank test. Wilcoxon tests were used to compare measurements between two groups(e.g., the 49 HRGs expressions between normal and cancer, TMB, stromal/immune/ESTIMATE scores between LHSG and HHSG). In comparison, Kruskal–Wallis tests were used for the comparison among three or more than three groups (e.g., the immune infiltration, immune-related function scores and the immune checkpoints expressions among three hypoxia clusters, HS among the three hypoxia clusters, three gene clusters, ten cell subpopulations). Correlation analysis was performed using the Spearman test. A *P* < 0.05 was considered statistically significant.

## Results

### Genetic variation in hypoxia-related genes among HNSCC patients

**Fig. 2:**
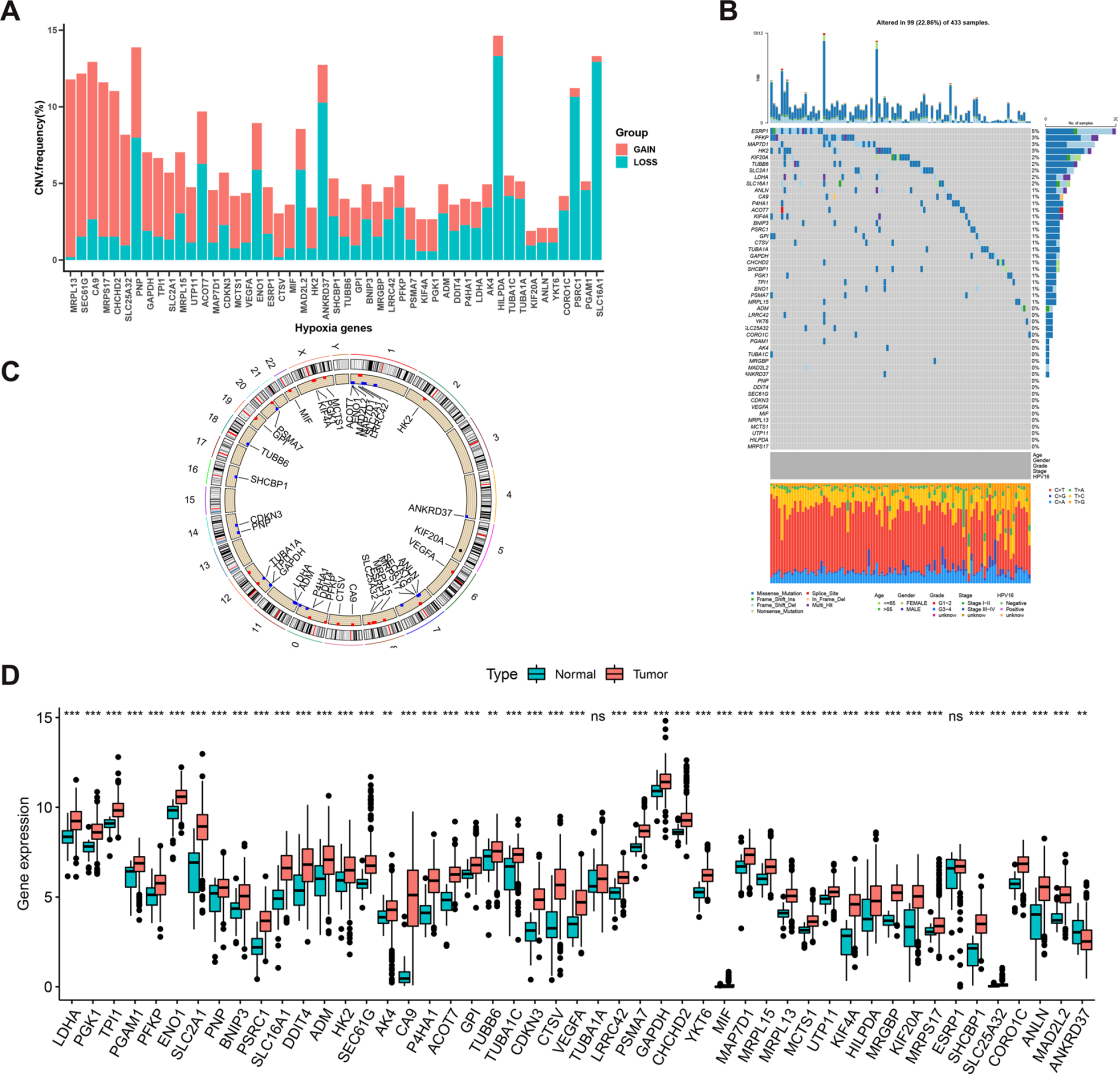
**A** CNV frequency of HRGs in TCGA-HNSCC cohort. **B** The mutation frequency of 49 HRGs in 433 patients with HNSCC from TCGA-HNSCC cohort. **C** The location of CNV alterations of HRGs on different chromosomes. **D** The expression of different HRGs between normal and tumor tissues (^ns^*P* > 0.05**,** *P < 0.05, **P < 0.01, ***P < 0.001). *CNV* copy number variation, *HNSCC* head and neck squamous cell carcinoma, *NS* not significant

Analysis showed that CNV was common among HRGs associated with HNSCC. While CNV is most often characterized by a reduction in copy number, more than half of the HRGs showed an increase in copy number (Fig. 2A). Ninety-nine (22.86%) of the 433 samples were found to contain HRG mutations, of which *ESRP1* was the most frequently mutated gene (Fig. 2B). CNV was also detected on these HRGs (Fig. 2C). Gene expression in normal and cancerous tissues was assessed and CNV was shown to affect HRG expression in HNSCC. While *ANKRD376* expression was significantly lower in cancer than in normal tissues, all other HRGs, except *TUBA1A* and *ESRRP1*, were significantly higher in cancer tissues (Fig. 2D). In addition, most HRGs were highly expressed in tumor tissues, suggesting that they may contribute to the occurrence and development of HNSCC.

### Stratification of HNSCC based on hypoxia-related gene sets

**Fig. 3:**
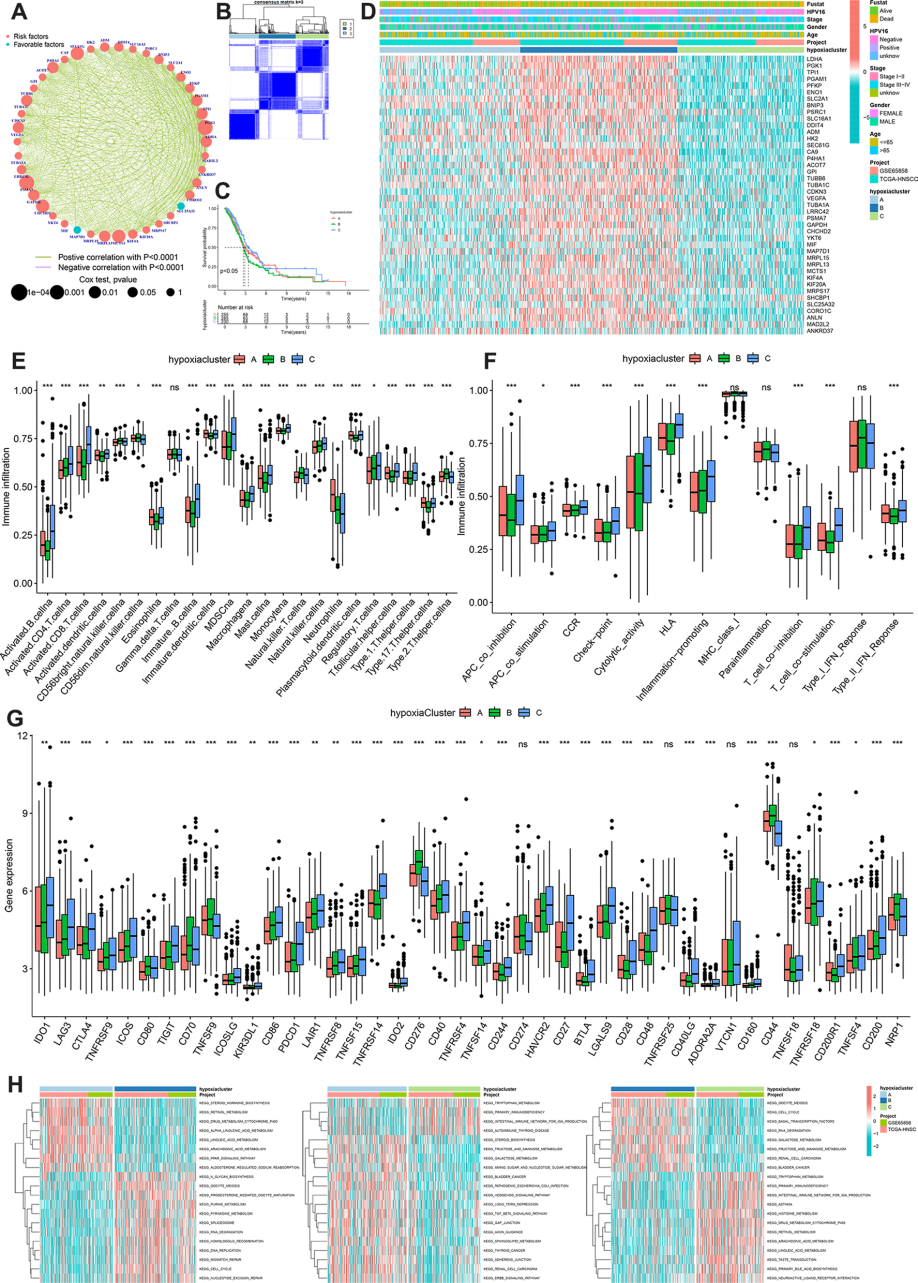
**A** The interaction between HRGs in HNSCC. The degree of HRG significance is represented by the size of the circle. **B** The consensus matrix for all HNSCC samples demonstrated clustering stability. Three subtypes were determined for all samples (k = 3). **C** OS differences among the three hypoxia clusters A, B, and C. **D** HRG expressions in the different hypoxia clusters and associated clinical characteristics. **E** Infiltration of 23 immune cell types. **F** Association with immune-related functions. **G** IC expressions in the three hypoxia clusters (^ns^*P* > 0.05**,** *P < 0.05, **P < 0.01, ***P < 0.001). **H** Activation status of biological behaviors among the three hypoxia clusters. *HRGs* hypoxia-related genes, *HNSCC* head and neck squamous cell carcinoma, *OS* overall survival, *IC* immune checkpoint, *ssGSEA* single-sample gene set enrichment analysis, *GSVA* gene set variation analysis

Hypoxia-related gene expression profiling of 770 HNSCC samples was obtained from TCGA and GSE65858 datasets. Univariate COX and correlation analysis were used to explore the interactions, connections, and effects of these genes on disease prognosis. Of these, 40 HRGs were risk factors (HR > 1), and two were protective factors (HR < 1) for HNSCC prognosis (all *P* < 0.05). Except for a significant negative correlation between *HK2* and *MAD2L2* expression, other prognosis-related HRGs were positively correlated (Fig. 3A). This finding suggests that there is possible crosstalk between these HRGs, which may correlate with disease prognosis and tumor heterogeneity among HNSCC patients.

A consensus cluster analysis of 770 samples was conducted to further explore the relationship between patient prognosis and the hypoxic signature and three HNSCC subclasses were identified, defined as hypoxia clusters A, B, and C (Fig. 3B). Of the clusters, hypoxic cluster C was associated with the best prognosis, while hypoxia cluster B was linked to the worst (*P* < 0.05) (Fig. 3C). Heatmap results showed that hypoxia clusters C and B had the lowest and highest HRGs expression, respectively (Fig. 3D). In contrast, hypoxia cluster A had the second-best prognosis with intermediate HRGs expression. Collectively, these results suggested that higher HRG expression was associated with poor HNSCC patient prognosis and that HRGs may play a key role in the malignancy of this disease.

### Immune characteristics and biological behaviors of the three hypoxia clusters

To better understand differences in the tumor microenvironment (TME) associated with the three hypoxia clusters, ssGSEA was used to assess immune cell infiltration and function in each cluster. Significant differences in the infiltration of 23 immune cell types, except for gamma delta T cells, were observed among the three hypoxia populations(all *P* < 0.05) (Fig. 3E). The TME of hypoxia cluster C was highly infiltrated by immune cells, including immunosuppressive cells such as myeloid-derived suppressor cells (MDSCs), macrophages, mast cells, and regulatory T cells (Tregs). A previous study found that hypoxia was linked to tumor immunosuppression by affecting immune cell infiltration [37]. MDSCs and Tregs are shown to be very active in highly hypoxia TME [11]. An additional study found that hypoxia can promote the development of malignant macrophages that aid tumor development [38]. High infiltration of immunosuppressive cells was associated with the poor prognosis of patients with HNSCC, pancreatic adenocarcinoma, esophageal cancer, or colon cancer [39–41]. The ranking of several cells with anti-tumor effects, including CD4 + T cells, CD8 + T cells, and natural killer (NK) cells, from higher to lower infiltration, were hypoxia cluster C, hypoxia cluster B and hypoxia cluster A (*P* < 0.05), possibly explaining why patients with three hypoxia clusters had the different prognosis.

APC inhibition, APC stimulation, CCR, immune checkpoints, cytolytic activity, HLA, inflammation promotion, T cell inhibition, T cell stimulation, and type II IFN responses also differed among the three clusters (Fig. 3F), and each of these processes was highly enriched in hypoxia cluster C patients with HNSCC, while the parainflammation was highly enriched in hypoxia cluster A. Given the increased use of immune checkpoint inhibitor (ICI)-based therapies, differences in immune checkpoint (IC) gene expression were also compared among the three clusters. The expression of *CD274*, *TNFRSF25*, *VTCB1*, *TNFSF18* and other ICs differed considerably among the clusters (Fig. 3G). KEGG-related GSVA was conducted to explore differences in the biological behaviors of the three hypoxia clusters (Fig. 3H). Diverse signaling pathways associated with inflammation, ebb signaling pathway, pathogenic escherichia coli infection were significantly enriched in cluster A. Elevated RNA degradation, cell cycle activity, spliceosome function, nucleotide excision repair, and mismatch repair reflected the high degree of hypoxia in cluster B while signaling pathways related to immunity, such as primary immunodeficiency, and the immune network for IgA production was significantly enriched in cluster C.

Taken together, these results showed that immune activation is strong in hypoxia cluster C, the inflammatory response is strong in hypoxia cluster A, and the degree of hypoxia is especially high in hypoxia cluster B. Hypoxia cluster A, Band C is the ‘immune-inflamed’, ‘immune-cold’ and ‘immune-hot’ phenotype, respectively. The consistency of the immune and prognostic profiles among the three groups supported this classification method as scientific and reasonable.

### Stratification of HNSCC based on differentially expressed genes

To identify the potential biological behavior of each hypoxia phenotype, 5,258 DEGs were identified in hypoxia cluster A vs. B, 4,113 DEGs were identified in hypoxia cluster A vs. C, and 6,915 DEGs were identified in hypoxia cluster B vs. C, respectively, using the ‘limma’ R package. An additional 688 overlapping DEGs were obtained through intersection analysis.

**Fig. 4:**
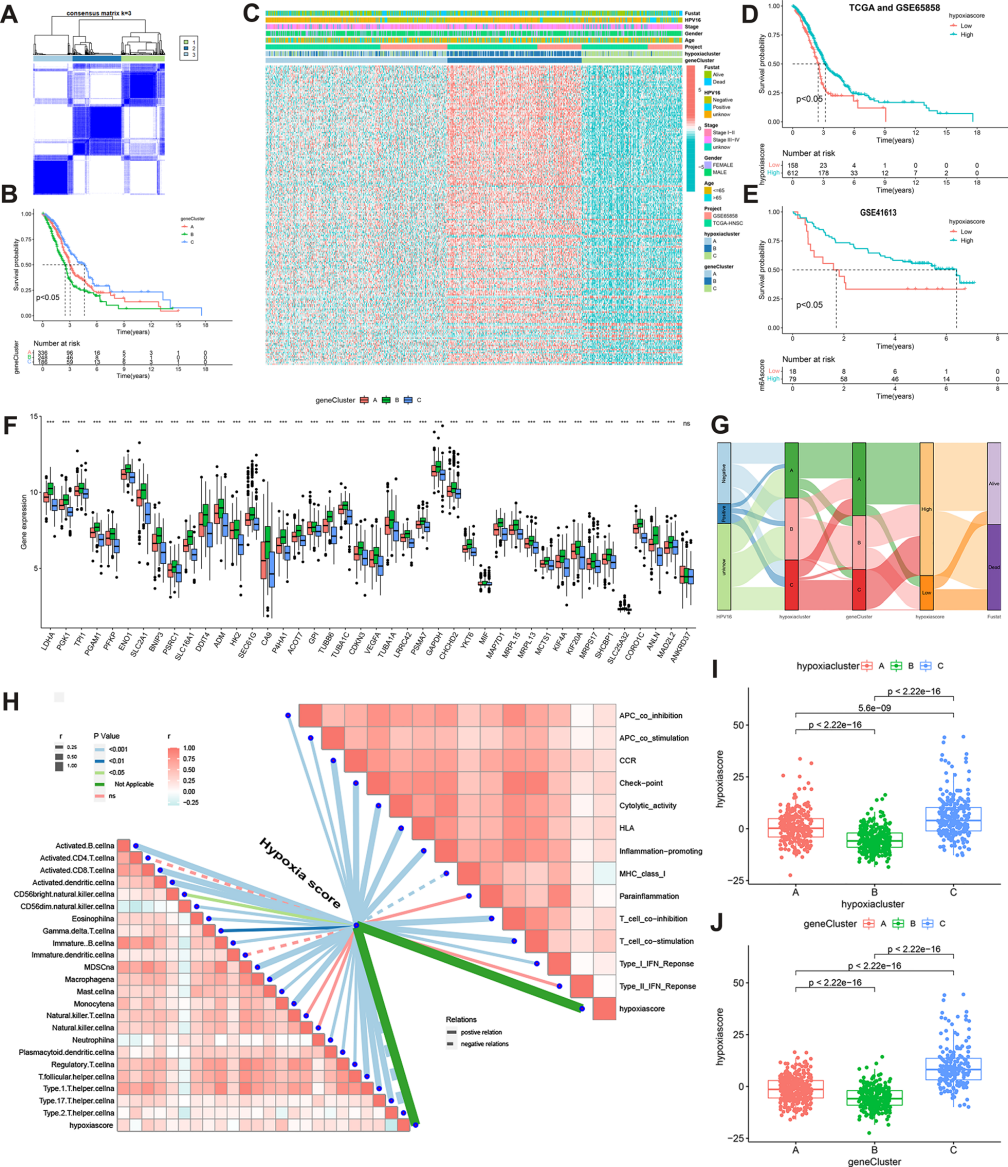
**A** Consensus matrixes for 770 HNSCC samples based on the DEGs among the three hypoxia clusters. Three subtypes were determined for all samples (k = 3). **B** OS differences among the three gene clusters. **C** Expression of hypoxia-related DEGs in different hypoxia clusters and gene clusters. **D–E** OS differences between HHSG and LHSG using a combination of TCGA-HNSCC and GSE65858, GSE41613 data. **F** Expression of different HRG in normal and tumor tissues (^ns^*P* > 0.05, *P < 0.05, ** *P* < 0.01, *** *P* < 0.001). **G** Sankey diagram showing the distribution of patients with HPV16, hypoxia clusters, gene clusters, HS, and survival status. **H** Correlations between HS, immune-related functions, and the abundance of each immune cell in 770 HNSCC samples. **I** Differences in the HS among the three hypoxia clusters. **J** Differences in the HS among the three gene clusters. *HNSCC* head and neck squamous cell carcinoma, *DEGs* differentially expressed genes, *OS* overall survival, *TCGA* The Cancer Genome Atlas, *HRG* hypoxia-related gene, *HHSG* high hypoxia score group, *LHSG* low hypoxia score group, *HS* hypoxia score

To further identify other phenotypic differences caused by hypoxic patterns, the overlapping DEGs were used to stratify patients into three DEG-related hypoxia clusters (gene clusters A–C) using an unsupervised cluster (Fig. 4A). Survival analyses showed that patients in gene cluster B had the worst prognosis (*P* < 0.001) (Fig. 4B). Most patients in gene clusters A, B, and C corresponded to hypoxic group clusters A, B, and C, respectively (Fig. 4C). Except for *ANKRD37*, the expression of all 48 HRGs differed significantly among the different gene clusters (Fig. 4F). Thus, the gene clusters corresponded well with the hypoxia-related clusters.

Considering the heterogeneity and complexity of individual hypoxia patterns, hypoxia patterns were quantified in all HNSCC patients using PCA and the results were expressed as an HS. After obtaining the HS optimal cutoff value(−7.012175) using the ‘maxstat’ R package (version:0.7–25) based on the TCGA and GSE65858 database, patients with HS values ≥ the optimal cutoff value were defined as the high HS group (HHSG) while those with HS values < the optimal cutoff value were defined as the low HS group (LHSG) [42]. Patients in the HHSG had a better prognosis than those in the LHSG using TCGA, GSE65858 (*P* < 0.05) (Fig. 4D) and GSE41463 (*P* < 0.05) data (Fig. 4E). Thus, HS has strong predictive power for the prognosis of patients with HNSCC.

To determine the relationship between HPV16 infection status, hypoxia clusters, gene clusters, and HS in each patient, HPV16 positive, negative, and unknown patients were classified into three hypoxia clusters and then divided into three gene clusters. Most patients in gene clusters A and C belonged to HHSG, while most patients in gene cluster B belonged to LHSG (Fig. 4G).

To further explore the correlation between immune cell infiltration in the TME and HS, immune cell infiltration and immune cell function scores were calculated in each sample using ssGSEA and a correlation analysis was conducted using the HS (Fig. 4H). There was a significant positive correlation between the HS and both immune cell infiltration and function. In addition, significant differences in the HS were observed among the three hypoxia clustering modes. Hypoxia cluster C had the highest median HS values, while hypoxic cluster B had the lowest median HS values (all *P* < 0.001) (Fig. 4I). Similar results were found in gene clusters C and B (all *P* < 0.001) (Fig. 4J). In summary, patients with hypoxia cluster C, gene cluster C and HHSG had the best prognosis, whereas those with hypoxia cluster B, gene cluster B, and LHSG had the worst prognosis. These results not only demonstrate the consistency of the predictive effect but also suggest that quantifying hypoxia status can help to predict patient prognosis and assess their immune-related characteristics.

### Hypoxia score combined with tumor mutation burden and stromal/immune/Estimate Scores could further refine the prognosis of HNSCC patients

**Fig. 5:**
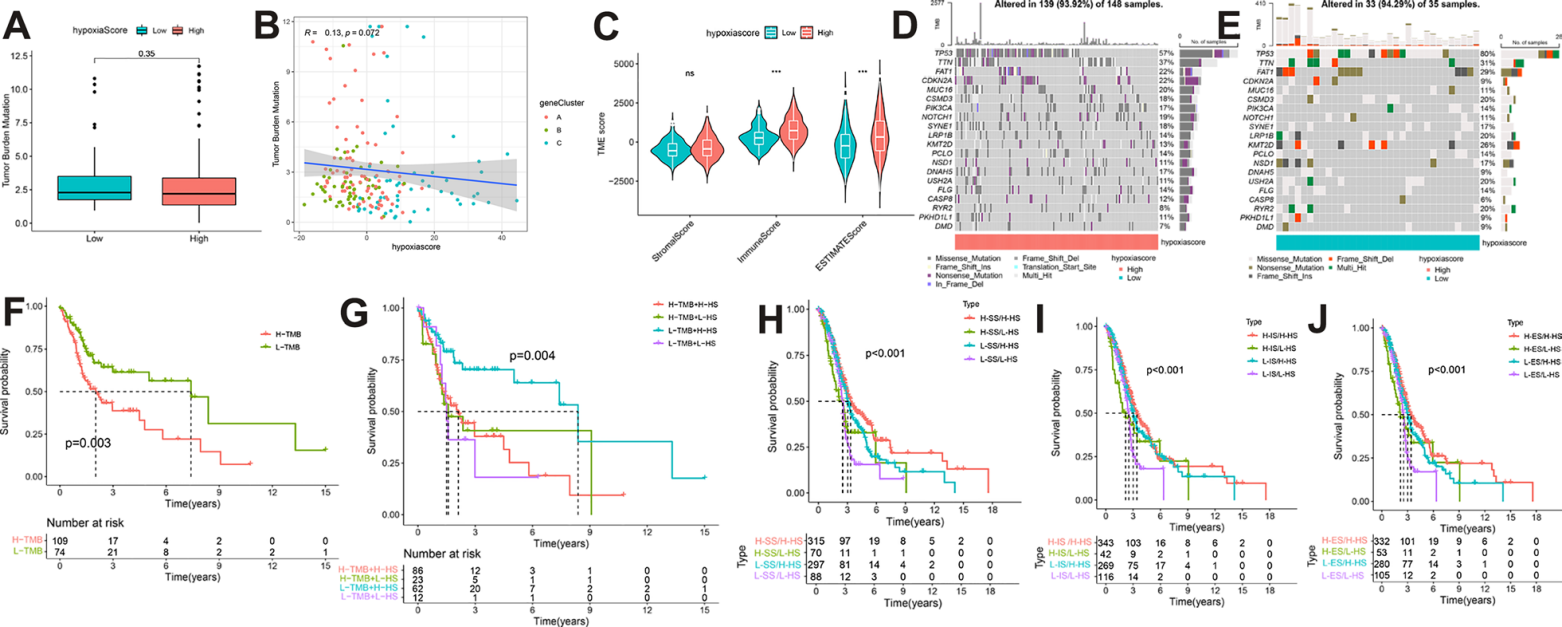
**A** Differences in TMB between the HHSG and LHSG. **B** The correlation between HS and TMB. **C** Differences in stromal, immune, and ESTIMATE scores between the HHSG and LHSG (^ns^*P* > 0.05**,** ***P < 0.001). **D, E** The top 20 driver genes with the highest mutation frequency in the **(D)** HHSG and **E** LHSG. **F** Differences in OS between the TMB subgroups. **G–J** OS differences stratified by TMB and HS, **H** stromal scores and HS, **I** immune scores and HS, and **J** ESTIMATE scores and HS. *TMB* tumor mutation burden, *HHSG* high hypoxia score group, *LHSG* low hypoxia score group, *OS* overall survival, *HS* hypoxia score

Tumor mutation burden (TMB) is a key criterion for successful immunotherapy [43].Thus, a series of analyses were conducted on somatic mutations associated with HNSCC. No differences in TMB were observed between the HHSG and LHSG groups (*P* > 0.05) and there was no significant association between the HS and TMB (*P* > 0.05) (Fig. 5A, B). The low HRG mutation frequency may account for these findings. Given that TMB is a prognostic factor in many types of cancer, a role for TMB and HS in HNSCC prognostic status was explored [44]. Prognosis was shown to be significantly higher among patients in the low TMB group than in the high TMB group (*P* < 0.05) (Fig. 5F). A German multi-center retrospective study found similar results [45]. Further results of the stratified prognostic analysis showed that patients with HHSG and low TMB had a large survival advantage (Fig. 5G). Since the frequency of HRG mutations in HNSCC is not high, a particular gene mutation in HNSCC was assessed and the top 20 driver genes with the highest mutation frequency were visualized. TP53 had the highest mutation frequency in both groups, followed by TTN (Fig. 5D, E).

The TME is critical to understanding the tumorigenesis and progression of many cancer types. Thus, stromal/immune/Estimate scores were calculated based on the ESTIMATE algorithm and the relationship between these scores and the HS was explored in patient prognosis. Findings showed that the immune/Estimate scores were higher in the HHSG than in the LHSG (all *P* < 0.05) (Fig. 5C). Next, patients were divided into high and low groups based on the median values of their stromal/immune/Estimate scores. The stratified prognostic analysis showed that those with a combination of HHSG and high stromal/immune/Estimate scores had a significant survival advantage (Fig. 5H–J). These data suggest that HS is a good prognostic indicator and that its combination with TMB and stromal/immune/Estimate scores can help to further refine patient prognosis.

### Hypoxia scores have a great potential to predict the efficacy of different therapies

**Fig. 6:**
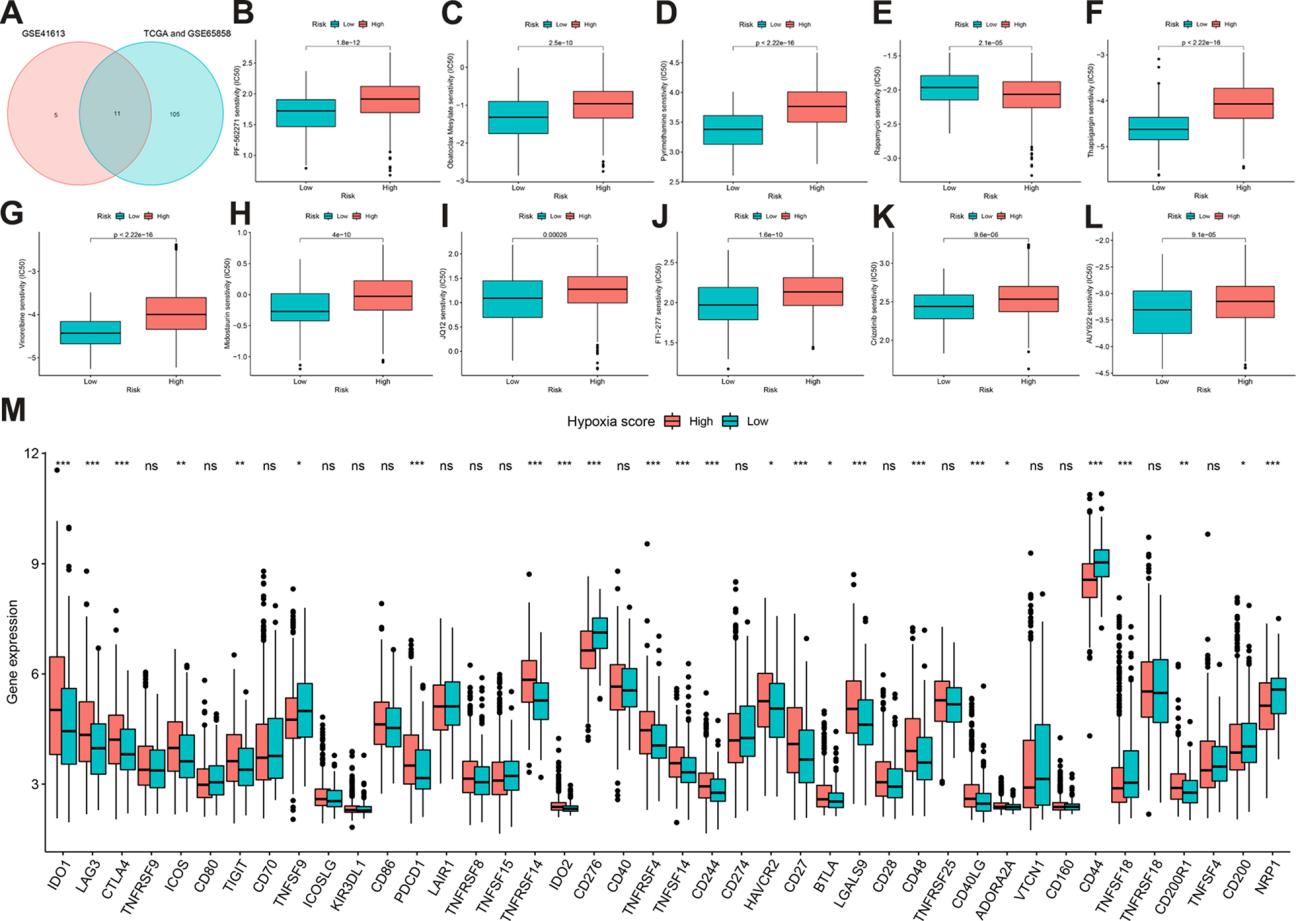
**A** The intersections of different sensitive TI between TCGA-HNSCC, GSE65858, and GSE41613. **B–L** The median IC50 of different TI in the HHSG and LHSG. The median IC50 was significantly lower for the LHSG than the HHSG for **B** PF-56227, **C** Obatoclax Mesylate, **D** Pyrimethamine, **E** Rapamycin, **G** Vinorelbine, **H** Midostaurin, **I** JQ12, **J** FTI-277, **K** Crizotinib, and **L** AUY922. The LHSG had a significantly higher median IC50 in the LHSG than the HHSG for (**F**) Thapsigargin. **M** Differences in IC gene expression between the HHSG and the LHSG (^ns^*P* > 0.05, *P < 0.05, **P < 0.01, ***P < 0.001). *TI* targeted inhibitor, *TCGA* The Cancer Genome Atlas, *HNSCC* head and neck squamous cell carcinoma, *HHSG* high hypoxia score group, *LHSG* low hypoxia score group, *IC* immune checkpoint

**Fig. 7:**
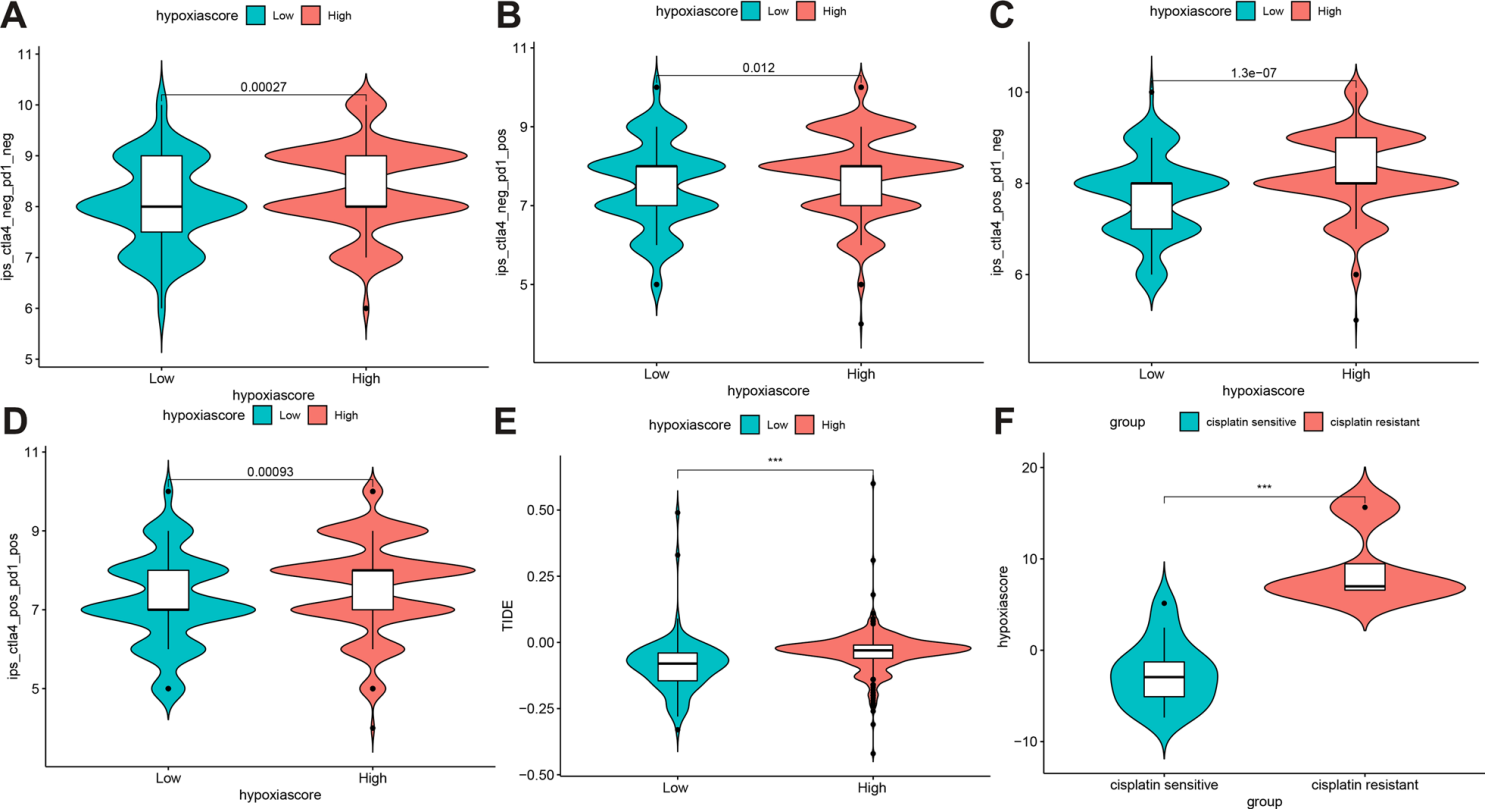
**A–D** Differences in the IPS between the HHSG and LHSG. The **A** IPS, **B** IPS-CTLA4, **C** IPS-PD1/PD-L1/PD- L2, and **D** IPS-PD1/PD-L1/PD-L2 + CTLA4 were significantly higher for the HHSG than the LHSG. **E** TIDE scores between the HHSG and LHSG (***P < 0.001). **F** HS between the cisplatin-sensitive and cisplatin-resistant groups. *IPS* Immunophenoscores, *HHSG* high hypoxia score group, *LHSG* low hypoxia score group, *TIDE* Tumor Immune Dysfunction and Exclusion

To assess the value of HS in predicting the clinical efficacy of HNSCC, the sensitivity of different drugs among patients in the HHSG and LHSG were analyzed using TCGA, GSE41613, and GSE65858 data. Using intersection analysis, 11 drugs were obtained with median IC50 values that differed between the two groups (Fig. 6). While the median IC50 was lower for the HHSG than the LHSG for rapamycin, the median IC50 was significantly higher for the HHSG than the LHSG for the other 10 drugs (all *P* < 0.001).*IDO1*, *LAG3*, *GTLA4*, *ICOS*, *TIGIT*, *PDCD1*, *TNFRSF8*, *TNFRSF14*, *IDO2*, *CD40*, *TNFRSF4*, *TNFSF14*, *CD244*, *HAVCR2*, *CD27*, *BTLA*, *LGALS9*, *CD48*, *CD40LG*, *ADORA2A*, and *CD200R1* had higher expression in the HHSG than in the LHSG (all *P* < 0.05), while *TNFSF9*, *CD270*, *CD274, CD44*, *TNFSF18*, *CD200*, *NPR1* had lower expression (Fig. 6M). Studies have demonstrated the ability of IPS to predict the immunotherapy response of patients with colon cancer and uterine corpus endometrial carcinoma [30, 46]. Thus, differences in IPS were assessed between HHSG and LHSG. The IPS (Fig. 7A), IPS-PD1/PD-L1/PD-L2 (Fig. 7B), IPS-CTLA4 (Fig. 7C), IPS-PD1/PD-L1/PD-L2 + CTLA4 (Fig. 7D), and TIDE scores (*P* < 0.05) (Fig. 7E) were significantly higher in the HHSG than in the LHSG (all *P* < 0.05). Meanwhile, the HS was significantly higher for the cisplatin-resistant group than for the cisplatin-sensitive group (*P* < 0.05) (Fig. 7F). Thus, HS has a strong ability to predict the effects of targeted drugs, immunotherapies, and chemotherapeutics.

### The key gene identified based on hypoxia scores

**Fig. 8:**
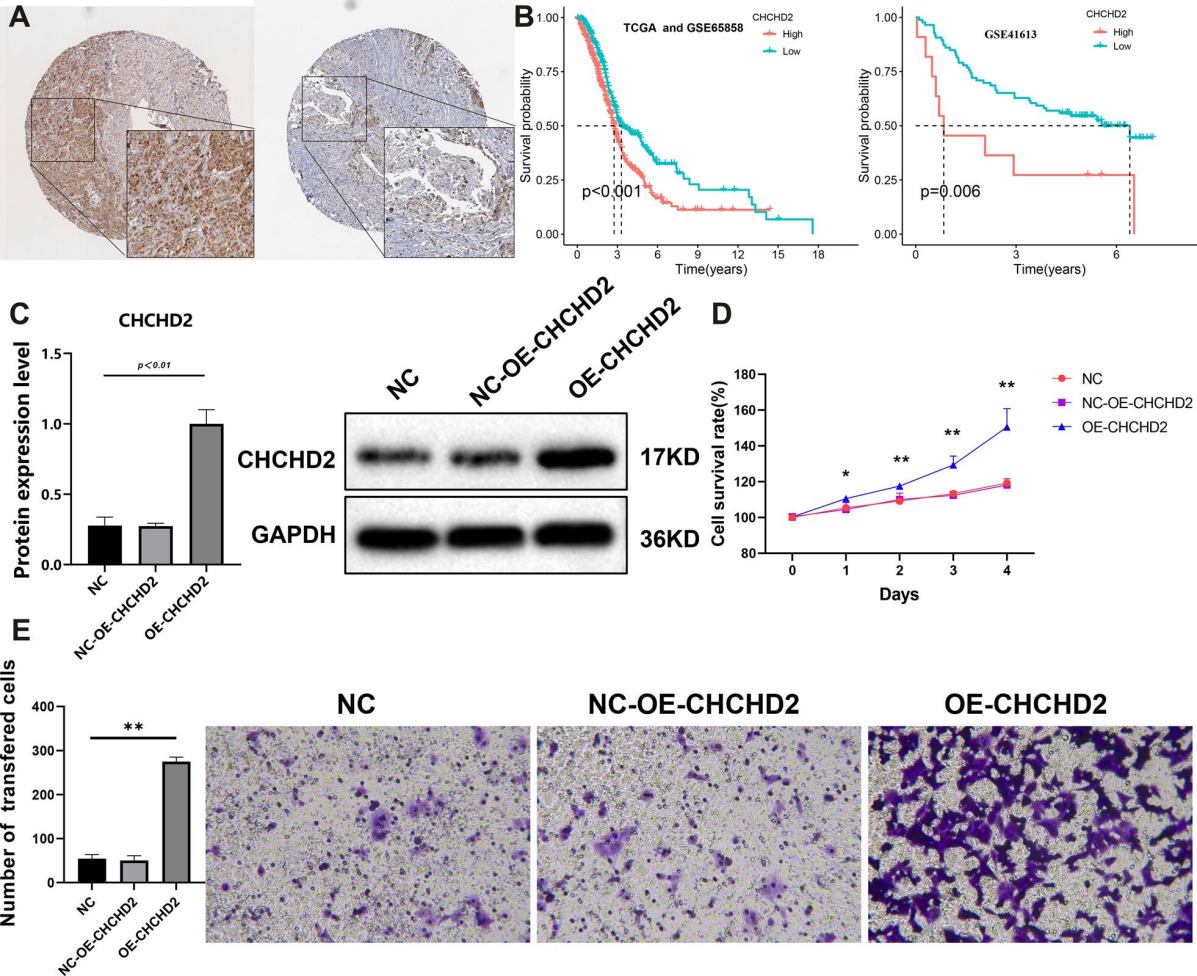
**A** Immunohistochemistry staining of the CHCHD2. Left to right: HNSCC and normal tissue. **B** Kaplan–Meier survival curve of CHCHD2 in HNSCC. The protein expression **C** and CCK-8 **D** and transwell assay **E** of CHCHD2 in vitro assays. ^**^P < 0.01.*OE* overexpression, *NC* negative control, *CCK* cell counting kit

As WGCNA is a powerful tool to find key genes that are highly correlated to corresponding modules, we adopted it to identify the key genes with high correlations with HS. Firstly, number 8 was chosen as the soft threshold (Additional file 1:Figure S1A). Subsequently, we obtained 9 modules (Additional file 1: Figure S1B). Thetwo modules, including black and brown,had significant positive correlations with HS, while the other four modules(green, blue, turquoise, grey) had negative correlations with HS(all *P* < 0.001, Additional file 1: Figure S1C). We selected the black and brown modules for further screening. After the intersection between module genes and HRGs was performed, *CHCHD2* was finally identified as a key gene. A previous study found that over-expression of *CHCHD2* could promote the expression of HIF-1α to adapt the hypoxia microenvironment in non-small cell lung cancer [47]. However, the exact mechanisms between *CHCHD2 *and hypoxia in HNSCC is still not clear. Then we further explored the function of this gene in HNSCC. Based on the HPA database, *CHCHD2* was highly expressed in HNSCC tissue and lowly expressed in normal tissue (Fig. 8A). Meanwhile, patients with higher expression of *CHCHD2* had a poorer prognosis than the patients with lower expression (all *P* < 0.05, Fig. 8B). Besides, in vitro experiments showed that overexpression of the *CHCHD2* promoted the migration and invasion of HNSCC cells (Fig. 8C–E). Taken together, it seems worthwhile to further explore the specific roles of *CHCHD2* in hypoxia-driven HNSCC progression.

### Single-cell sequencing data revealed the mechanism of different immune cells in hypoxia

**Fig. 9:**
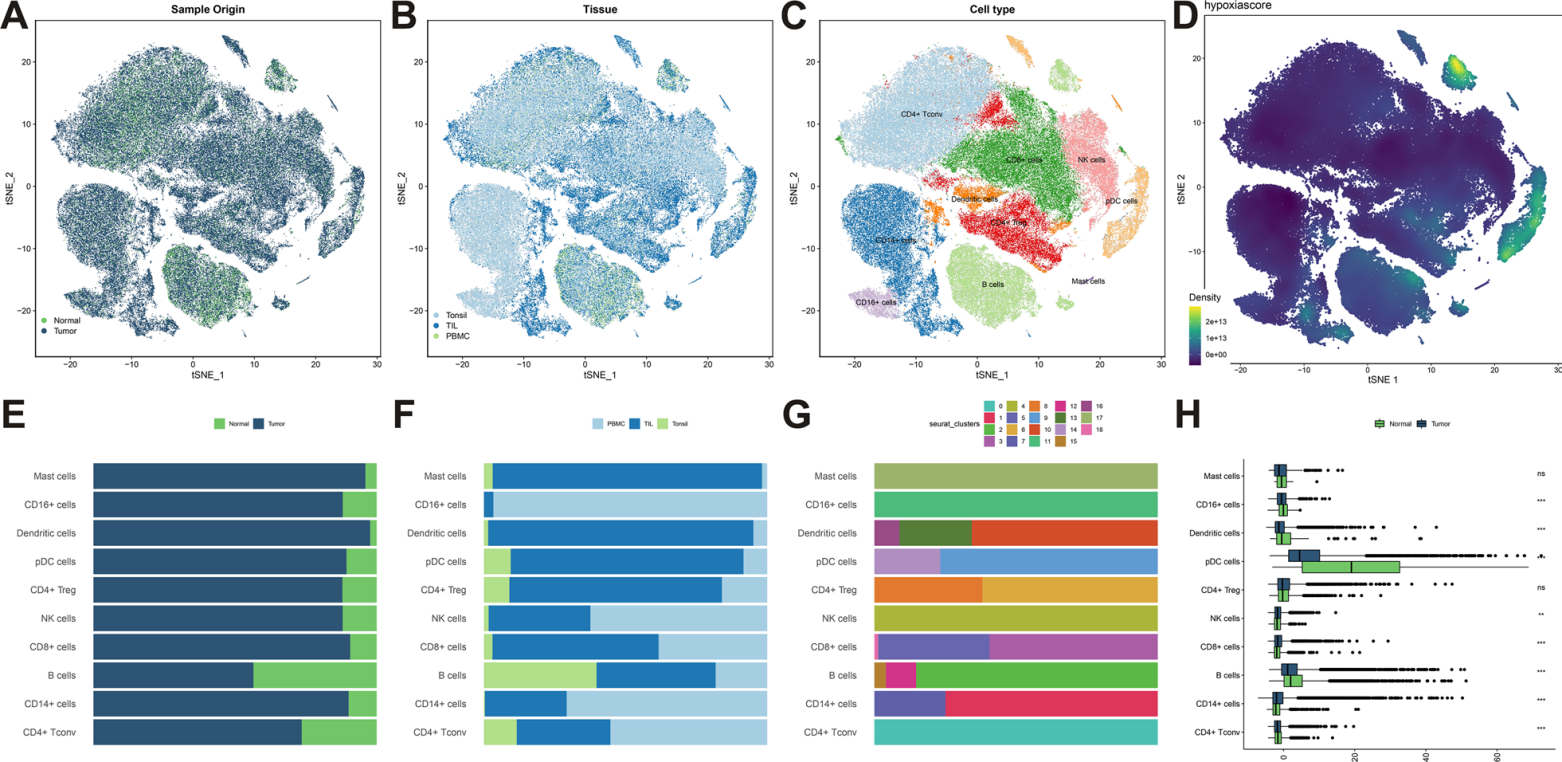
**A-D** tSNE of the 121,347 cells profiled and color-coded by **A** sample type, **B** tissue type, **C** cell type, and **D** HS. The bar diagram shows the fraction of cells originating from **E** normal and tumor samples, **F** PBMC, TIL, and tonsil **G** 19 clusters. **H** Box plots of the HS in different cell types. *HS* hypoxia score, *PBMC* peripheral blood mononuclear cell, *TIL* tumor-infiltrating lymphocytes

A single-cell sequencing-based analysis was performed to further reveal the hypoxia mechanism in different immune cells. Immune cell samples from five tonsil tissues, thirty-one peripheral blood mononuclear cells (PBMC), and 26 tumor-infiltrating lymphocytes (TIL) were obtained from the single-cell sequencing dataset, GSE139324. A total of 121,347 cells from 62 samples were assessed, of which 99,261 were from patients with HNSCC and 22,086 were from the normal population (Fig. 9A–B, Additional file 2: Figure S2A–B). Subsequently, 19 clusters were presented by graph-based t-distributed stochastic neighbor embedding (TSNE) (Additional file 2: Figure S2C), and the top five markers of the 19 clusters were presented in dotplot(Additional file 2: Figure S2D). Based on the expression levels of marker genes those obtained from previous literature (Additional file 2: Figure S2E), ten cell subpopulations were identified and shown in TSNE (Fig. 9C). Besides, the fraction of different cell subsets in normal and tumor, PBMC, tonsil, and TILs,19 clusters were shown in Fig. 9E–G, respectively. Each cell was assigned an HS using the same PCA method (Fig. 9D). The HS of the CD16 + cells, Dendritic cells,pDC cells, B cells, and CD4 + Tconv cells in the tumor tissue were lower than those in normal tissue (*P* < 0.05), while NK cells, CD8 + cells, and CD14 + cells vice versa (*P* < 0.05) (Fig. 9H). The HS of CD4 + Tconv cells was significantly lowest, while pDC cells was highest in the ten cell types (Additional file 3: Figure S3A, B). The GSVA analysis was used to further clarify the biological functions of different cell subpopulations (Additional file 3: Figure S3D). GSVA-based biological analysis showed that hypoxia pathways were active in the CD16 + cells, and Dendritic cells, and inhibited in the CD4 + Tconv cells, B cells, and CD8 + cells. Collectively, these results may suggest potential targets and mechanisms of the hypoxia in the tumor immune microenvironment (TIME) of HNSCC.

### Cell–cell communication in HNSCC

**Fig. 10:**
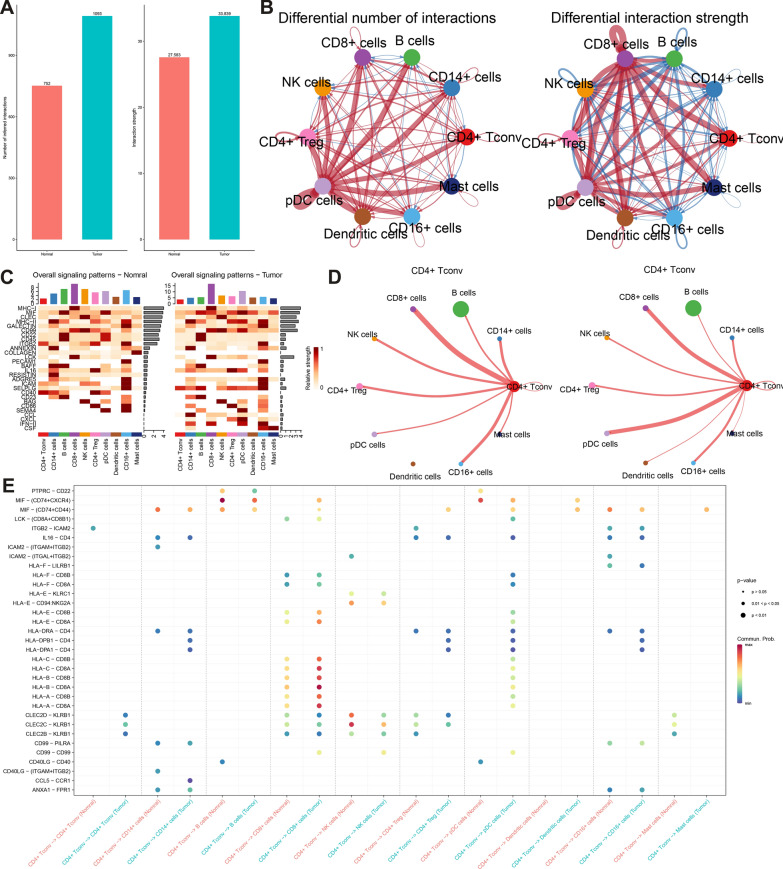
**A** Bar plot showing the interaction number and strength between normal and HNSCC. **B** Diagrams displaying the interaction number and strength in cell clusters. **C** Heatmap depicting signals contributing the most to the overall signaling pathways in normal and HNSCC. **D** The number of ligand-receptor between CD4 + T conv cells and other cell subpopulations. **E** Comparison of the significant ligand-receptor pairs between CD4 + T conv cells and other cells

To further investigate the interaction network in the TIME in HNSCC, we used ‘Cellchat’ R package to uncover alterations in cell crosstalk between normal tissues populations and HNSCC. Compared with the normal tissues, the overall number and strength of interactions were all increased (Fig. 10A), and the number and strength of most of the interactions among all the immune cells were increased and stronger, respectively (Fig. 10B). The above results indicate TIME is a complex milieu. Then we further compared the signals patterns between tissue from normal populations and HNSCC. The overall signal patterns of normal and HNSCC were cleanly presented in Fig. 10C. For example, the SELPLG signal strength was ranked as the first in the CD4 + T conv cells derived from HNSCCs. Next, the focus was set on the CD4 + T conv cells as its lowest HS among all the immune cells and hypoxia-related function. Compared with normal tissues, we found the most significant changes number of ligand-receptor between the CD4 + T conv cells were pDC cells in the HNSCC, and the number of ligand-receptor among these immune cells was increased (Fig. 10D). Furthermore, we analyzed the receptor ligands that may regulate communication between CD4 + T conv cells and other immune cells. For example, CD4 + T conv communicated with pDC cells using the *CD74* + *CXCR4*, *CD74* + *CCD44*, which was not found in normal tissues (Fig. 10E), suggesting the role of CD4 + T conv cell-derived *CD74* in the progress of HNSCC. A previous study found that *CD74* and migration inhibitory factor (*MIF*) plays a vital role in HNSCC progression [48]. Besides, Zhu et al. [49] revealed that *MIF* bind to *CD74/CXCR2*, *CD74/CXCR4* could play a vital role in the hypoxia-induced tumor growth in HNSCC. Thus, the association of hypoxia and the communication between CD4 + T conv cells and pDC cells deserves further investigation.

### Prognostic role of HLA-DPA1 and CD4 in HNSCC

**Fig. 11:**
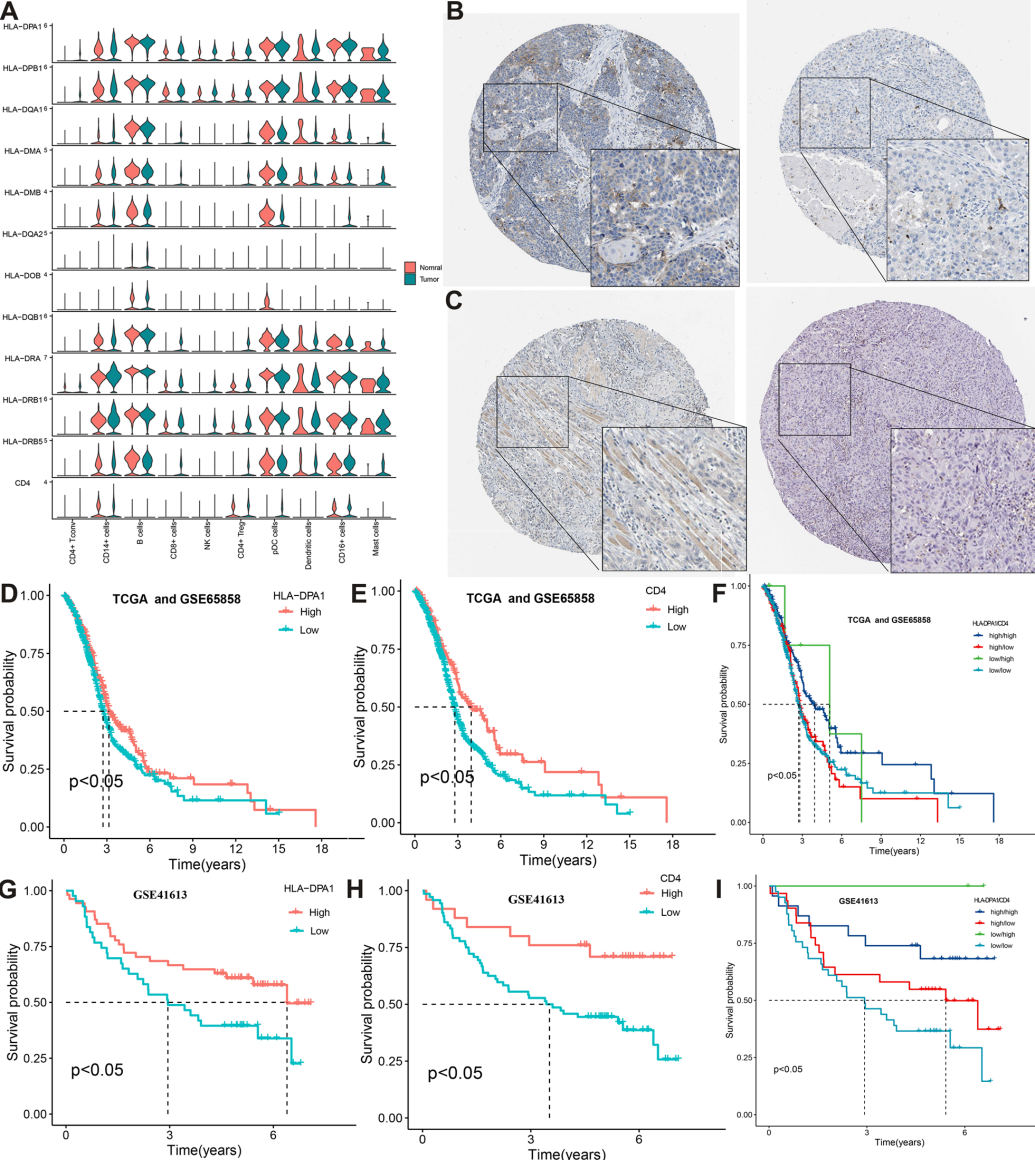
**A** Expression distribution of MHC-II signaling genes at tissue from normal and HNSCC. Immunohistochemistry staining of the **B** HLA-DPA1 and **C** CD4, left to right, HNSCC and normal tissue. Kaplan–Meier survival curve of HLA-DPA1 and CD4 in HNSCC from **D–F** TCGA-HNSCC, GSE65858 **G–I** GSE41613. *MHC* major histocompatibility complex

As CD4 + T Conv cells communicated with pDC cells mainly using the MHC-II pathway (Fig. 10E), we further investigated the MHC class II (MHC II) pathway gene expression in different immune cells between normal tissues and HNSCC. Compared with the normal tissues, the expression of *HLA-DPA1* was increased significantly in the CD4 + T Conv cells, while receptor *CD4* was also increased significantly in the pDC cells (Fig. 11A), explaining the reason for the construction of the MHC-II pathway between CD4 + T Conv cells and pDC cells in the tissues from HNSCC. *HLA-DPA1,* an MHC II molecule, is primarily expressed on professional antigen-presenting cells and is responsible for presenting exogenous peptide antigens to CD4 + T cells [50]. *CD4* is the marker gene of CD4 + T cells. Given the close relations between MHC II, *CD4* and immune, we adopted the TIMER database to analyze the relationship between *HLA-DPA1* and *CD4* expression and representative markers of different immune infiltrating cells in patients with HNSCC, respectively. The adjusted results based on tumor purity showed a significant correlation between *HLA-DPA1, CD4,* and all immune cell types (Table 4) (all *P* < 0.05). To validate this result, different algorithms were adopted using the GSCA website and ssGSEA algorithms. All immune cells except mucosal-associated invariant T were significantly associated with *HLA-DPA1* and *CD4* expression in HNSCC patients (Table 5) (all *P* < 0.05), and the ssGSEA algorithms also revealed that all 23 immune cells infiltration abundance had significant associations with both the expression of *HLA-DPA1 and CD4* (Additional file 3: Figure S3C) (all *P* < 0.05). These results demonstrate the tight link between *HLA-DPA*, *CD4,* and HNSCC-specific immunity. Additionally, recent studies suggest that tumor-specific MHC-II may also function as a tumor suppressor, and expression is associated with favorable outcomes in lung cancer and pan-cancer patients [51–53]. Besides, CD4 + T cells play an anti-tumor role in human bladder cancer [54], and the higher levels of CD4 + T cells infiltration had a better prognosis than the lower levels in sarcoma patients [55]. Therefore, it is reasonable to believe that *HLA-DPA1* and *CD4* may play antitumor effects in HNSCC. We further analyzed the association between *HLA-DPA1* and *CD4* and prognosis. Survival analysis defined patients with *HLA-DPA1* and *CD4* mRNA expression levels  ≥ optimal cutoff value (of 7.7662 and 6.175818, respectively) as the high group and those with expression < optimal cutoff value as the low group. Patients in the high group of *HLA-DPA1* had higher OS than those in the low group in TCGA, GSE65858 (*P* < 0.05), and GSE41613 datasets (*P* < 0.05) (Fig. 11D–E), while the same results were also validated in *CD4*(Fig. 11G–H)*.* Besides, we divided the patients into *HLA-DPA1* high/*CD4* high and the other three groups. The survival result showed patients with low expression of *HLA-DPA1* and high expression of *CD4*, high expression of *HLA-DRA1* and high expression of *CD4* had better prognoses than the other two groups (Fig. 11F, I). Altogether, the above results support the hypothesis that *HLA-DPA1*/*CD4* axis plays antitumor effects in HNSCC. The HPA database was used to compare *HLA-DRA* protein expression in normal and HNSCC tissues. Interestingly*, HLA-DRA* was low expressed, while *CD4* was weakly expressed in HNSCC tissues, both *HLA-DPA1*/*CD4* were not detected in the normal tissues (Fig. 11B–C). The positive correlation of the *HLA-DPA1*/*CD4* expression with some immune cells with anti-tumor effects such as NK cells may explain this phenomenon.

**Table 4:** Correlation analysis between HLA-DPA1 and CD4 and gene markers of different immune cells in HNSCC from the TIMER database

Cell type	Gene markers	HLA-DPA1				CD4			
		None		Purity		None		Purity	
		Cor	p-value	cor	p-value	Cor	p-value	Cor	p-value
CD8 + T	CD8A	8.06E-01	0.00E + 00	−2.77E-01	4.03E-10	7.48E-01	1.75E-89	−2.77E-01	4.03E-10
	CD8B	7.65E-01	1.45E−101	−2.42E-01	5.06E-08	7.15E-01	2.38E-78	−2.42E-01	5.06E-08
T cell (general)	CD3D	8.01E-01	0.00E + 00	−2.98E-01	1.35E-11	7.46E-01	1.47E-88	−2.98E-01	1.35E-11
	CD3E	8.47E-01	9.59E−145	−2.99E-01	1.17E-11	8.43E-01	2.18E-134	−2.99E-01	1.17E-11
	CD2	8.50E-01	0.00E + 00	−2.85E-01	1.15E-10	8.36E-01	3.54E-130	−2.85E-01	1.15E-10
B cell	CD19	4.77E-01	5.74E−31	−2.61E-01	4.33E-09	5.05E-01	2.74E-33	−2.61E-01	4.33E-09
	CD79A	4.71E-01	3.47E−30	−2.28E-01	3.05E-07	5.12E-01	2.50E-34	−2.28E-01	3.05E-07
Monocyte	CD86	7.73E-01	0.00E + 00	−2.95E-01	2.26E-11	8.45E-01	2.22E-135	−2.95E-01	2.26E-11
	CD115 (CSF1R)	8.33E-01	6.76E−136	−3.04E-01	4.98E-12	9.39E-01	5.83E-230	−3.04E-01	4.98E-12
TAM	CCL2	5.50E-01	0.00E + 00	−2.58E-01	5.98E-09	6.13E-01	2.64E-52	−2.58E-01	5.98E-09
	CD68	4.52E-01	0.00E + 00	−1.72E-01	1.24E-04	5.68E-01	1.75E-43	−1.72E-01	1.24E-04
	IL10	5.57E-01	7.92E−44	−3.13E-01	1.21E-12	6.28E-01	1.74E-55	−3.13E-01	1.21E-12
M1 macrophage	INOS (NOS2)	2.55E-01	3.57E−09	7.08E-02	1.16E-01	2.96E-01	2.09E-11	7.08E-02	1.16E-01
	IRF5	3.14E-01	3.02E−13	−1.43E-03	9.75E-01	3.82E-01	1.44E-18	−1.43E-03	9.75E-01
M2 macrophage	COX2 (PTGS2)	-1.53E-01	4.45E−04	9.98E-02	2.67E-02	-1.55E-01	5.32E-04	9.98E-02	2.67E-02
	CD163	6.89E-01	0.00E + 00	−2.86E-01	1.02E-10	8.07E-01	2.37E-114	−2.86E-01	1.02E-10
	VSIG4	6.22E-01	0.00E + 00	−2.57E-01	7.42E-09	7.53E-01	3.02E-91	−2.57E-01	7.42E-09
	MS4A4A	7.38E-01	5.15E−91	−2.87E-01	8.35E-11	8.38E-01	1.73E-131	−2.87E-01	8.35E-11
Neutr-ophils	CD66b (CEACAM8)	5.92E-02	1.77E−01	3.42E-02	4.48E-01	4.51E-02	3.18E-01	3.42E-02	4.48E-01
	CD11b (ITGAM)	6.00E-01	2.39E−52	−1.37E-01	2.27E-03	7.20E-01	7.13E-80	−1.37E-01	2.27E-03
	CCR7	6.52E-01	1.71E−64	−3.22E-01	2.23E-13	7.38E-01	9.46E-86	−3.22E-01	2.23E-13
NK cell	KIR2DL1	3.81E-01	1.69E−19	−9.34E-02	3.81E-02	3.44E-01	4.23E-15	−9.34E-02	3.81E-02
	KIR2DL3	5.10E-01	6.99E−36	−1.36E-01	2.56E-03	4.69E-01	2.57E-28	−1.36E-01	2.56E-03
	KIR2DL4	6.02E-01	7.92E−53	−1.83E-01	4.30E-05	4.95E-01	7.06E-32	−1.83E-01	4.30E-05
	KIR3DL1	4.47E-01	4.78E−27	−1.44E-01	1.35E-03	4.27E-01	2.70E-23	−1.44E-01	1.35E-03
	KIR3DL2	5.74E-01	4.40E−47	−1.47E-01	1.05E-03	5.56E-01	2.63E-41	−1.47E-01	1.05E-03
	KIR3DL3	2.33E-01	7.00E−08	−8.59E-02	5.68E-02	2.05E-01	4.56E-06	−8.59E-02	5.68E-02
	KIR2DS4	3.47E-01	2.92E−16	−1.48E-01	9.93E-04	3.32E-01	4.09E-14	−1.48E-01	9.93E-04
Dend-ritic cell	HLA-DPB1	9.57E-01	0.00E + 00	−3.02E-01	7.78E-12	8.70E-01	1.09E-152	−3.02E-01	7.78E-12
	HLA-DQB1	8.14E-01	6.55E−125	−2.28E-01	3.28E-07	7.03E-01	1.07E-74	−2.28E-01	3.28E-07
	HLA-DRA	9.74E-01	0.00E + 00	−2.99E-01	1.16E-11	8.51E-01	1.33E-139	−2.99E-01	1.16E-11
	BDCA-1	5.37E-01	2.59E−40	−2.57E-01	7.19E-09	6.25E-01	1.07E-54	−2.57E-01	7.19E-09
	BDCA-4	4.59E-01	1.61E−28	−2.07E-01	3.38E-06	5.65E-01	6.16E-43	−2.07E-01	3.38E-06
	CD11c	6.45E-01	1.01E−62	−2.83E-01	1.53E-10	7.89E-01	7.01E-106	−2.83E-01	1.53E-10
	T-bet (TBX21)	7.88E-01	9.07E−112	−2.44E-01	4.20E-08	7.68E-01	2.85E-97	−2.44E-01	4.20E-08
Th1	STAT4	6.52E-01	0.00E + 00	−2.61E-01	3.96E-09	6.94E-01	6.30E-72	−2.61E-01	3.96E-09
	STAT1	6.26E-01	0.00E + 00	−2.40E-01	7.06E-08	5.29E-01	5.97E-37	−2.40E-01	7.06E-08
	IFN-y(IFNG)	7.09E-01	5.81E−81	−2.34E-01	1.49E-07	5.79E-01	2.19E-45	−2.34E-01	1.49E-07
	TNF-a(TNF)	2.28E-01	1.47E−07	−1.27E-01	4.90E-03	2.26E-01	4.19E-07	−1.27E-01	4.90E-03
Th2	GATA3	4.56E-01	3.72E−28	−2.27E-01	3.63E-07	4.93E-01	1.61E-31	−2.27E-01	3.63E-07
	STAT5A	6.20E-01	8.53E−57	−1.32E-01	3.28E-03	6.43E-01	9.08E-59	−1.32E-01	3.28E-03
	STAT6	2.90E-01	1.73E−11	6.90E-02	1.26E-01	3.25E-01	1.31E-13	6.90E-02	1.26E-01
	IL13	4.10E-01	1.52E−22	−1.54E-01	5.78E-04	4.22E-01	1.06E-22	−1.54E-01	5.78E-04
Tfh	BCL6	8.87E-02	4.28E−02	1.75E-01	8.98E-05	1.69E-01	1.57E-04	1.75E-01	8.98E-05
	IL21	5.20E-01	2.03E−37	−1.75E-01	9.02E-05	5.12E-01	2.50E-34	−1.75E-01	9.02E-05
Th17	STAT3	3.84E-01	0.00E + 00	−1.69E-02	7.08E-01	4.09E-01	2.82E-21	−1.69E-02	7.08E-01
	IL17A	2.85E-01	3.45E−11	−1.02E-01	2.40E-02	2.74E-01	6.59E-10	−1.02E-01	2.40E-02
Treg	FOXP3	7.93E-01	0.00E + 00	−2.53E-01	1.18E-08	8.70E-01	9.39E-153	−2.53E-01	1.18E-08
	CCR8	6.88E-01	2.64E−74	−2.34E-01	1.39E-07	7.89E-01	4.56E-106	−2.34E-01	1.39E-07
	STAT5B	3.76E-01	6.17E−19	−5.12E-02	2.56E-01	4.91E-01	2.65E-31	−5.12E-02	2.56E-01
	TGFb(TGFb1)	4.60E-02	2.95E−01	−1.42E-01	1.55E-03	1.36E-01	2.39E-03	−1.42E-01	1.55E-03
T-cell -exhau-stion	PD-1(PDCD1)	8.20E-01	7.03E−128	−2.68E-01	1.57E-09	7.74E-01	1.45E-99	−2.68E-01	1.57E-09
	CTLA4	7.66E-01	0.00E + 00	−3.10E-01	2.04E-12	7.69E-01	1.14E-97	−3.10E-01	2.04E-12
	LAG3	7.67E-01	2.04E−102	−2.37E-01	9.98E-08	6.97E-01	7.88E-73	−2.37E-01	9.98E-08
	TIM-3(HAVCR2)	8.68E-01	0.00E + 00	−2.77E-01	3.81E-10	9.26E-01	2.09E-209	−2.77E-01	3.81E-10
	GZMB	7.22E-01	0.00E + 00	−2.64E-01	2.68E-09	6.14E-01	2.30E-52	−2.64E-01	2.68E-09

**Table 5:** Correlation between HLA-DPA1 and CD4 expression and the infiltration of immune cells in HNSCC from GSCA

Cancer	Symbol	Cell type	Cor	p_value	Fdr
HNSC	CD4	Bcell	0.125229401	0.002840681	0.00709747
HNSC	CD4	CD4_T	0.498597147	6.63574E-37	2.34323E-35
HNSC	CD4	CD4_naive	−0.32641244	1.61664E-15	2.27551E-13
HNSC	CD4	CD8_T	0.487711864	3.70562E-35	2.41381E-33
HNSC	CD4	CD8_naive	−0.27974378	1.23245E-11	3.86398E-10
HNSC	CD4	Central_memory	0.399107546	4.69625E-23	1.75049E-21
HNSC	CD4	Cytotoxic	0.648867218	6.05612E-69	6.66107E-67
HNSC	CD4	DC	0.264792464	1.55096E-10	1.81922E-09
HNSC	CD4	Effector_memory	−0.348009036	1.47439E-17	3.6235E-16
HNSC	CD4	Exhausted	0.430230051	6.61951E-27	3.14239E-25
HNSC	CD4	Gamma_delta	0.350506267	8.36139E-18	1.3932E-16
HNSC	CD4	InfiltrationScore	0.774932647	1.8103E-114	6.1759E-112
HNSC	CD4	MAIT	−0.079904273	0.057456885	0.102199733
HNSC	CD4	Macrophage	0.623132464	3.43057E-62	7.75849E-60
HNSC	CD4	Monocyte	0.026849971	0.52380974	0.648854849
HNSC	CD4	NK	0.452353693	6.74773E-30	3.80443E-28
HNSC	CD4	NKT	−0.069342829	0.099341311	0.161500841
HNSC	CD4	Neutrophil	−0.734956861	3.26494E-97	7.73137E-95
HNSC	CD4	Tfh	0.61809823	6.07528E-61	5.07399E-59
HNSC	CD4	Th1	0.400553951	3.17398E-23	1.31996E-21
HNSC	CD4	Th17	−0.522771785	5.12345E-41	7.99417E-39
HNSC	CD4	Th2	0.151716151	0.000291823	0.001462605
HNSC	CD4	Tr1	0.526495547	1.11241E-41	2.11779E-39
HNSC	CD4	iTreg	0.578673885	6.65288E-52	2.15983E-49
HNSC	CD4	nTreg	0.132102751	0.001634437	0.004873403
HNSC	HLA-DPA1	Bcell	0.160458983	0.000126143	0.000439536
HNSC	HLA-DPA1	CD4_T	0.390194718	5.0338E-22	5.91479E-21
HNSC	HLA-DPA1	CD4_naive	−0.341193739	6.75694E-17	1.51115E-14
HNSC	HLA-DPA1	CD8_T	0.571758553	1.92463E-50	2.54861E-48
HNSC	HLA-DPA1	CD8_naive	−0.353117464	4.59558E-18	6.00057E-16
HNSC	HLA-DPA1	Central_memory	0.317720549	9.66095E-15	1.05282E-13
HNSC	HLA-DPA1	Cytotoxic	0.718927306	3.78606E-91	9.64631E-89
HNSC	HLA-DPA1	DC	0.170909511	0.000043664	0.000157248
HNSC	HLA-DPA1	Effector_memory	−0.142157787	0.000694338	0.001711443
HNSC	HLA-DPA1	Exhausted	0.527673099	6.83588E-42	8.82004E-40
HNSC	HLA-DPA1	Gamma_delta	0.399847288	3.84448E-23	1.39931E-21
HNSC	HLA-DPA1	InfiltrationScore	0.6560327	6.07356E-71	6.57251E-69
HNSC	HLA-DPA1	MAIT	−0.069344284	0.099334199	0.161528417
HNSC	HLA-DPA1	Macrophage	0.482428439	2.47987E-34	1.81509E-32
HNSC	HLA-DPA1	Monocyte	−0.170950179	0.000043478	0.000248193
HNSC	HLA-DPA1	NK	0.545950055	2.77657E-45	4.17066E-43
HNSC	HLA-DPA1	NKT	−0.11742411	0.005156123	0.012552302
HNSC	HLA-DPA1	Neutrophil	−0.72886484	7.40981E-95	1.60371E-92
HNSC	HLA-DPA1	Tfh	0.636744419	1.10534E-65	1.08002E-63
HNSC	HLA-DPA1	Th1	0.549856923	4.91135E-46	6.21734E-44
HNSC	HLA-DPA1	Th17	−0.493278817	4.82249E-36	4.78163E-34
HNSC	HLA-DPA1	Th2	0.188362098	6.42744E-06	0.000064815
HNSC	HLA-DPA1	Tr1	0.327139515	1.38843E-15	1.57711E-14
HNSC	HLA-DPA1	iTreg	0.493931466	3.78772E-36	3.23048E-34
HNSC	HLA-DPA1	nTreg	0.127127138	0.002444997	0.006899699

## Discussion

As hypoxia plays a critical role in the progression of HNSCC, Wang et al. [56] found that two hypoxia clusters had a significant difference in the OS and two hypoxia-immune clusters had a significant difference in the therapeutic efficacy, which is similar to that of our study. To precisely explore the heterogeneity of tumors, the current study obtained three hypoxia-related clusters that act as different immune related-phenotypes, providing more subtypes to predicting clinical outcomes and guiding personalized clinical treatment. As the algorithm for obtaining the hypoxia cluster could not quantify the hypoxia features of each HNSCC sample, we further constructed a system called HS using the PCA algorithm to explore the relationship between hypoxia and HNSCC further. The current findings also suggested that the HS can reliably predict HNSCC outcomes. There is evidence that hypoxia could reduce the survival, cytolytic and migrating activities of effector cells such as CD4 + cells, CD8 + cytotoxic T cells, natural killer cells, and natural killer-like cells [57]. Then the HS had positive correlations with the infiltration of immune cells with anti-tumor effects, such as CD8 + T cells and CD56 NK cells and may account for the HHSG having a better prognosis than the LHSG.

The current treatment of HNSCC has become increasingly diverse and individualized.Zandberg et al. [58] reported that hypoxia was increased in the anti-PD-1 resistant tumors of recurrent/metastatic patients with HNSCC. The current study also demonstrated that the expression of most ICs differed significantly between the HHSG and LHSG. Thus, targeting hypoxia-related signaling pathways may increase the efficacy of immunotherapies such as immune checkpoint blockade, and enhance the immune response. The bioinformatics-based study found that the hypoxia‑derived gene model could guide the anti‑PD‑L1 treatment [56]. In addition, a previous study also found that hypoxia targeting multimodal therapy could enhance the sensitivity of chemotherapy among HNSCC patients [59]. The current findings not only showed similar results but also demonstrated that HS can be used to predict the efficacy of 11 drugs and responsiveness to more immunotherapy drugs, including anti-CTLA4, anti-PD‑L1 PD1/PD-L1/PD-L2 + CTLA4, anti-PD1/PD-L1/PD- L2.

Nowadays, the integrating analysis of bulk RNA-seq and single-cell RNA-seq to explore the molecular cellular mechanisms of gene patterns has attracted the attention of researchers [60, 61]. Our study also uncovered the potential roles of hypoxia in the TIME of HNSCC. The HS of CD4 + T Conv was lowest among all the immune cells. Some different expression genes between CD4 + T Conv cells and others were found to have close associations with hypoxia, such as *ISG15* and *PARK7*. *ISG15* was identified as a hypoxia- and immune-related gene signature that could predict clinical outcomes of patients with prostate [62]. *PARK7* had been experimentally shown to be an important mediator of hypoxia-induced cellular responses [63]. In addition, GSVA analysis suggested that hypoxia was suppressed in CD4 + T Conv cells. A deeper understanding of the specific roles played by CD4 + T Conv cells in hypoxia-driven tumor progression is therefore necessary. Next, we identified ligand-receptor HLA-DPA1/CD4 mediating cell crosstalk between CD4 + T conv cells and pDC cells. After a careful literature search, we found no studies on the prognostic aspects of CD4 in cancer. A previous study revealed that the expression level of HLA-DPA1 was higher in tumor tissues than that in normal tissues and patients with the high level of HLA-DPA1 had a good prognosis in skin cutaneous melanoma [64]. Similar results were also found in current study.

## Conclusion

The HS that was used to determine hypoxia patterns may serve as a biomarker to predict HNSCC prognosis and treatment responsiveness. The key gene *CHCHD2* acts as an oncogene and was verified by overexpression experiments. Moreover, we explored crosstalk between CD4 + Tconv cells and pDC cells, *HLA-DPA1*–*CD4* axis was identified as a unique interaction between the two immune cells. Thus, a novel and powerful tool was developed to aid the construction of hypoxia immunophenotypes, prediction of clinical outcomes, provision of individualized treatments, demonstration of immune landscape, and provided new therapeutic targets for immunotherapies in patients with HNSCC.

## Supplementary Information

**Additional file 1: Figure S1.** **(A)** Scale-free co-expression network. **(B)** The branches of the dendrogram. **(C)** The correlation between each gene module and HS. HS, hypoxia score.

**Additional file 2: Figure S2.** **(A-C)** tSNE of the 121,347 cells profiled and color-coded by **(A)** patients, **(B)** sample type, **(C)** clusters. C means healthy donor, T means patients with HNSCC; Cxp and Txp mean sample from PBMC. Dotplot showing the expression of top 5 marker genes in the **(D)** 19 clusters**(E)** 10 cell types.

**Additional file 3: Figure S3.** **(A-B)** The differences in HS among 10 cell types. **(C)** Correlations between expression of HLA-DPA1(left) and CD4(right) and the abundance of each immune cell infiltration in 770 HNSCC samples.** (D)** Differences in pathway activities scored per cell by GSVA in 10 cell types. HS, hypoxia score; GSVA, gene set variation analysis.

## Data Availability

The original data used for the current study were derived from public databases.

## Abbreviations

HNSCC: Head and neck squamous cell carcinoma
HS: Hypoxia score
HPV: Human papillomaviruses
QOL: Quality of life
TME: Tumor microenvironment
TIME: Tumor immune microenvironment
HIF1α: Hypoxia-inducible factor 1α
DCs: Dendritic cells
NK: Natural killer
pDC: Plasmacytoid dendritic cells
HHSG: High HS group
LHSG: Low HS group
TPM: Transcripts per kilobase million
MSigDB: Molecular signature database
ssGSEA: Single-sample gene set enrichment analysis
TCIA: The cancer immunome Atlas
TIDE: Tumor immune dysfunction and exclusion
PBMC: Peripheral blood mononuclear cell
TIL: Tumor-infiltrating lymphocytes
TIMER: Tumor immune estimation resource
GSCA: Gene set cancer analysis
