# Supplementary figures and images for: Multi-omics characterization of a scoring system to quantify hypoxia patterns in patients with head and neck squamous cell carcinoma
Source: J Transl Med. 2023 Jan 10;21:15. doi: 10.1186/s12967-022-03869-8 (PMC9830846; doi:10.1186/s12967-022-03869-8)

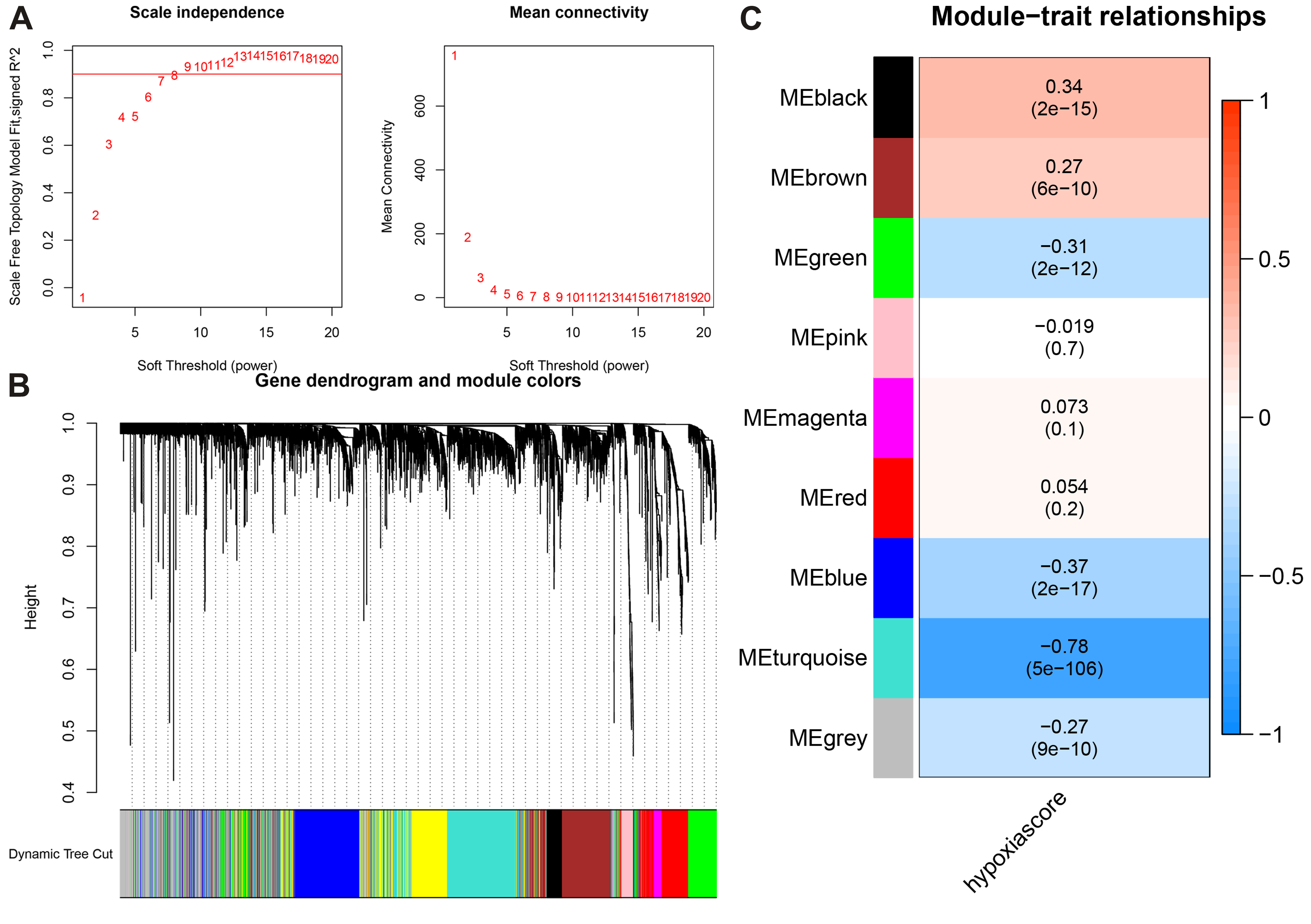

Supplement: Supplementary file 1 — Additional file 1: Figure S1. (A) Scale-free co-expression network. (B) The branches of the dendrogram. (C) The correlation between each gene module and HS. HS, hypoxia score. [file 12967_2022_3869_MOESM1_ESM.tif]

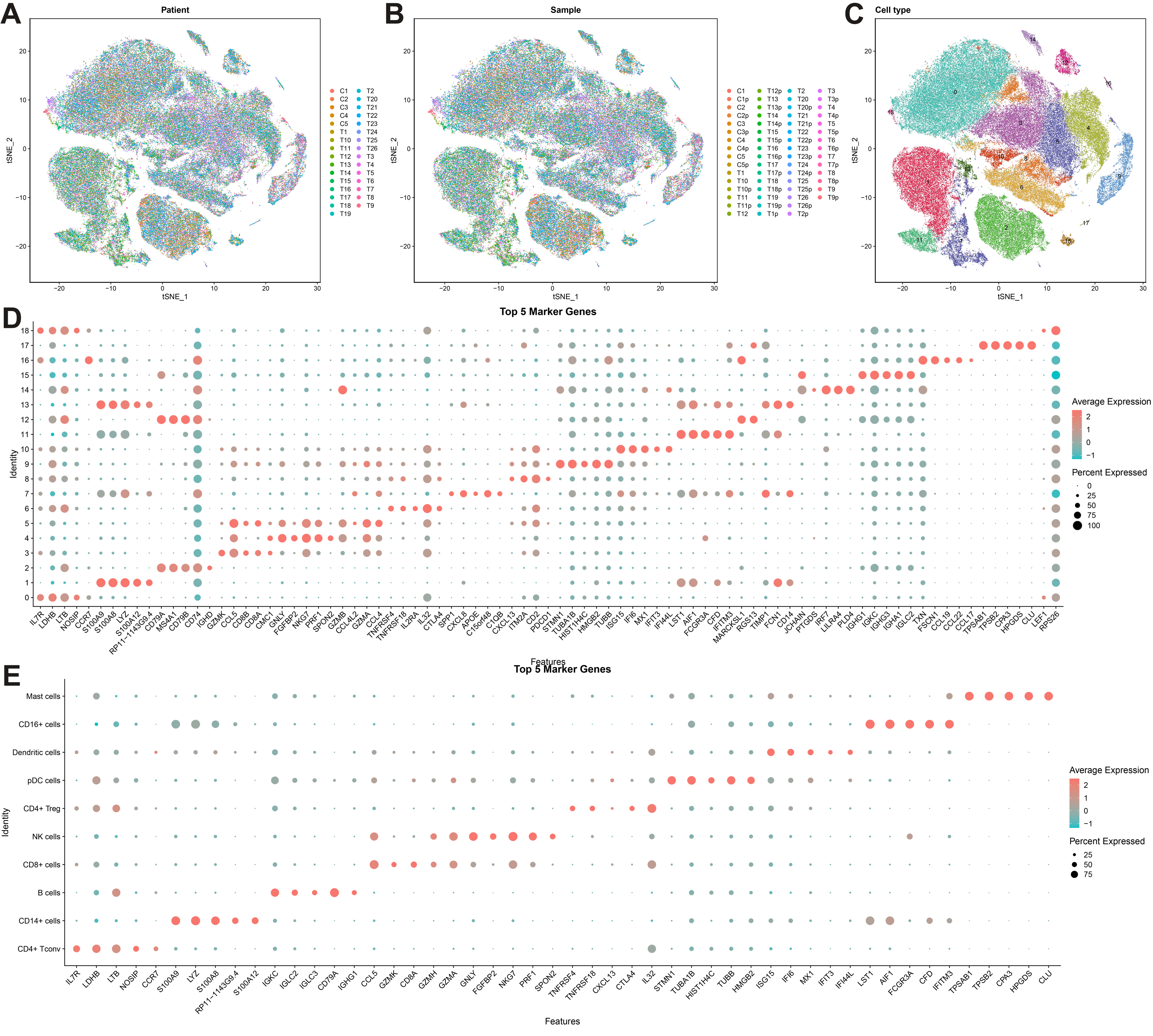

Supplement: Supplementary file 2 — Additional file 2: Figure S2. (A-C) tSNE of the 121,347 cells profiled and color-coded by (A) patients, (B) sample type, (C) clusters. C means healthy donor, T means patients with HNSCC; Cxp and Txp mean sample from PBMC. Dotplot showing the expression of top 5 marker genes in the (D) 19 clusters(E) 10 cell types. [file 12967_2022_3869_MOESM2_ESM.tif]

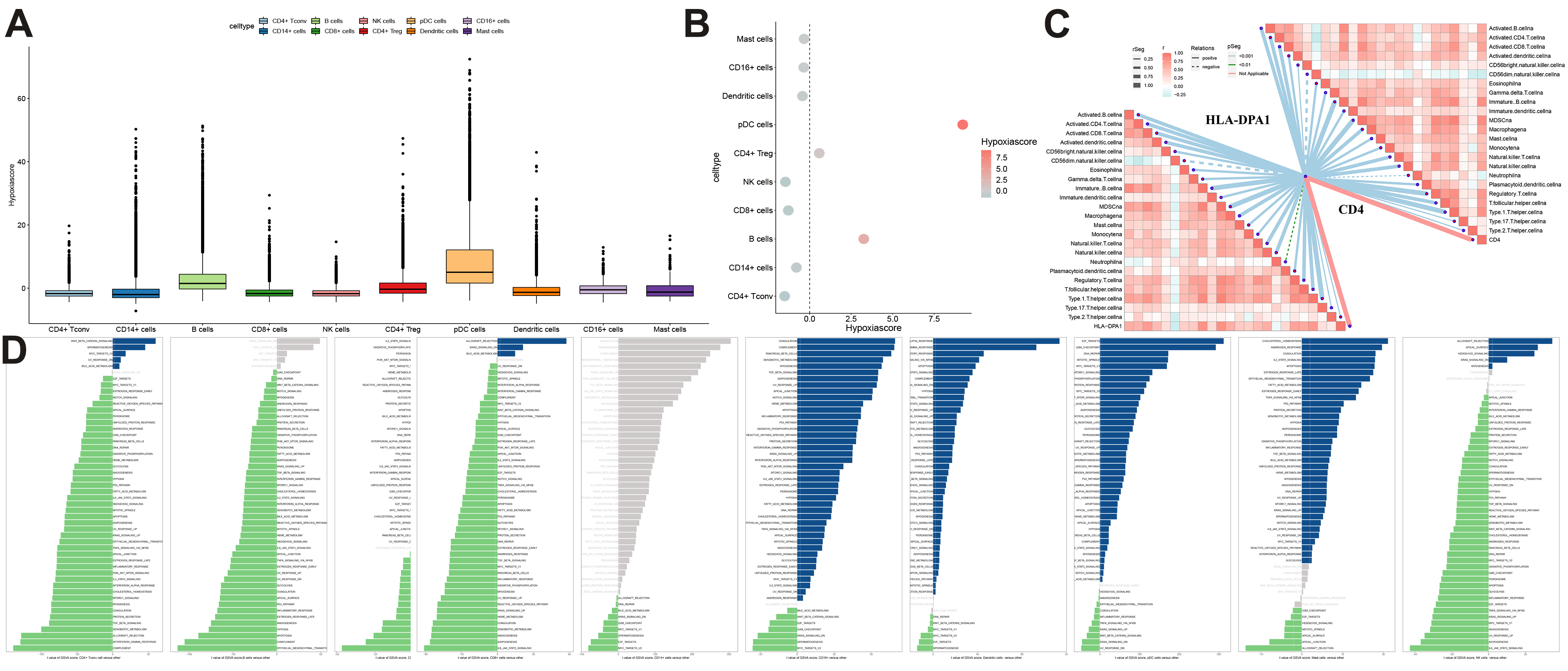

Supplement: Supplementary file 3 — Additional file 3: Figure S3. (A-B) The differences in HS among 10 cell types. (C) Correlations between expression of HLA-DPA1(left) and CD4(right) and the abundance of each immune cell infiltration in 770 HNSCC samples. (D) Differences in pathway activities scored per cell by GSVA in 10 cell types. HS, hypoxia score; GSVA, gene set variation analysis. [file 12967_2022_3869_MOESM3_ESM.tif]
